# Notch Signaling Regulates Muscle Stem Cell Homeostasis and Regeneration in a Teleost Fish

**DOI:** 10.3389/fcell.2021.726281

**Published:** 2021-09-28

**Authors:** Sami H. A. Sultan, Carlene Dyer, Robert D. Knight

**Affiliations:** ^1^Centre for Craniofacial and Regenerative Biology, King’s College London, Guy’s Hospital, London, United Kingdom; ^2^William Harvey Research Institute, Barts and The London School of Medicine and Dentistry, Queen Mary University of London, London, United Kingdom

**Keywords:** Notch, skeletal muscle, zebrafish, regeneration, satellite cell, DAPT

## Abstract

Muscle regeneration is mediated by the activity of resident muscle satellite cells (muSCs) that express Pax7. In mouse Notch signaling regulates muSCs during quiescence and promotes muSC proliferation in regeneration. It is unclear if these roles of Notch in regulating muSC biology are conserved across vertebrates or are a mammalian specific feature. We have therefore investigated the role of Notch in regulating muSC homeostasis and regeneration in a teleost fish, the zebrafish. We have also tested whether muSCs show differential sensitivity to Notch during myotome development. In an absence of injury Notch is important for preventing muSC proliferation at the vertical myoseptum. In contrast, Notch signaling promotes proliferation and prevents differentiation in the context of injury. Notch is required for the proliferative response to injury at early and later larval stages, suggesting it plays a similar role in regulating muSCs at developing and adult stages. Our results reveal a conserved role for Notch signaling in regulating muSCs under homeostasis and for promoting proliferation during regeneration in teleost fish.

## Introduction

Skeletal muscle, composed of myofibre bundles arranged into fascicles, is the largest organ by mass in the body and is essential for locomotion, breathing and posture. Muscle is highly regenerative and even fairly extensive injuries can be repaired. The regeneration of myofibres is performed in mammals by a tissue resident stem cell population called muscle satellite cells (muSCs). These are a resident stem cell that express Pax7 in mammals, frogs and fish and are localized beneath the myofibre cell membrane when quiescent (Bentzinger et al., 2012). Other genes expressed by muSCs in mice include c-Met and Pax3, although it is not clear whether expression is in all cells or reflects heterogeneity between muSCs (Webster and Fan, 2013; Der Vartanian et al., 2019). In response to injury of the muscle there is an initial influx of inflammatory cells and mobilization of tissue resident macrophages and fibroblasts. Subsequently the muSCs are activated and delaminate, migrate, proliferate and then differentiate to form new myofibres, or repair damaged myofibres through fusion.

The Notch pathway has been shown to be important for regulating muSCs in amniotes such as mice, humans, and chickens (Mourikis and Tajbakhsh, 2014). Activation of this signaling pathway results in transcriptional activation of the RBP-J transcription factor caused by nuclear translocation of the cell surface Notch receptor in response to binding of Delta or Jagged receptors presented on adjacent cells. Notch signaling has been shown to regulate muSC proliferation after injury and promotes expansion of the progenitor pool in mice (Brack et al., 2008). A conditional loss of the Notch-activated RBP-J transcription factor results in a depletion of muscle progenitor cells, impaired muscle growth and loss of adult satellite cells (Vasyutina et al., 2007). Another important function of Notch is to regulate quiescence of muSCs (Bjornson et al., 2012; Mourikis et al., 2012) as genetic inhibition of the pathway results in muSC activation, whereas over-activation prevents the exit from quiescence (Wen et al., 2012). In the chick Notch is known to regulate specification of muscle progenitor cells during embryogenesis (Delfini et al., 2000). In chick epithelial cells of the somites, Myf5 expression is induced by Delta1-expressing neural crest as it migrates past them thus activating myogenesis (Rios et al., 2011). Notch has also been shown to be activated in satellite cells of chick as a consequence of force-induced myofibre stretching and JAG-2 induction, highlighting the central role of Notch in regulating muSC activity in amniotes (Esteves de Lima et al., 2016). It is unclear whether Notch plays a similar role in regulating muSCs outside of the amniotes, although descriptions of deficiencies in fin muscle development of embryos treated with the Notch inhibitor DAPT, suggest myogenic progenitors require Notch for their development (Pascoal et al., 2013).

Similar to mice, muSCs in zebrafish are activated by injury and migrate to regenerate damaged muscles (Rowlerson et al., 1997; Knappe et al., 2015; Gurevich et al., 2016; Pipalia et al., 2016; Berberoglu et al., 2017). Most descriptions of muSCs in zebrafish have focused on cells that express *pax7* genes, although it is known that cells expressing *pax3*, *myf5*, and *met* also contribute to muscle formation and repair in early stage larvae at 3 days post-fertilization (dpf) (Gurevich et al., 2016; Nguyen et al., 2017; Ratnayake et al., 2021). Similar to descriptions from mice, zebrafish show developmental differences in the distribution and populations of pax7-expressing progenitor cells in muscle (Devoto et al., 2006; Buckingham, 2007; Buckingham and Relaix, 2007; Knappe et al., 2015). This implies that mechanisms important for regulation of pax7-expressing muSCs in early larval stages may change during development and therefore not reflect the adult condition. We and others have previously documented cell rearrangements of pax7 and pax3 expressing muSCs during early development (Hollway et al., 2007; Stellabotte et al., 2007; Nguyen et al., 2017; Roy et al., 2017). Such rearrangements reflect the movement of muscle progenitor cells to form the peripheral external cell layer (ECL) in the myotome, which subsequently move into the myotome to generate new myofibres (Devoto et al., 2006; Nguyen et al., 2017). Our earlier comparisons of muSC responses at 3 and 7 dpf showed that at later larval stages the pax7a-expressing muSCs are mostly quiescent with little contribution to growth, compared to earlier stages when dynamic and rapid movement of muSCs within the myotome correlated with their contribution to myofibre formation (Knappe et al., 2015). In adult animals, pax7a:eGFP and pax3a:egfp-expressing cells express pax7 protein and are localized laterally to the slow muscle portion of the myotome (Berberoglu et al., 2017). These cells reside beneath the basal lamina of the myofibres similar to muSCs described in frog and mammals (Mauro, 1961; Bentzinger et al., 2012; Ganassi et al., 2020) and contribute to myofibre repair after focal injury (Berberoglu et al., 2017). This implies that pax7a:eGFP expressing muSC progenitor cells become quiescent after early larval stages (from 6 dpf onward) and act as the resident stem cell population. Whether muSCs are functionally different after 6 dpf compared to earlier stages (at 3 dpf) is unclear. The majority of studies investigating muscle regeneration in zebrafish have used animals between 3 and 4 dpf as these are classified as non-protected by most national regulatory frameworks (Gurevich et al., 2016; Pipalia et al., 2016; Ratnayake et al., 2021). Extensive changes to the extracellular matrix and reorganization of muscle progenitor cells occur in the myotome between 4 and 7 dpf (van Eeden et al., 1996; Devoto et al., 2006; Roy et al., 2017). It is therefore important to determine whether the changing environment of the myotome alters responses of muSCs to injury and the signals regulating these.

In this work we have addressed a number of outstanding questions concerning the regulation of muSC responses to injury *in vivo* in zebrafish. We have investigated whether developmental stage affects the muSC responses to injury, performed power calculations for identifying changes to muSC number after injury and tested whether Notch signaling regulates muSC homeostasis and proliferation. We find that inhibition of Notch activity attenuates muSC proliferation, resulting in premature differentiation. Intriguingly, we observed that in an absence of Notch activity, proliferation of muSCs at the myosepta is increased in uninjured animals. This suggests that Notch is required to maintain muSC homeostasis at the myosepta, analogous to the role of Notch in maintaining quiescence of muSCs in mammals.

## Materials and Methods

### Fish Stocks and Maintenance

Adult zebrafish were maintained using a per standard protocol (Snider and Clegg, 1975). Embryos were collected and raised in E3 media at 28.5°C. 24 h post-fertilisation (hpf) embryos were transferred into E3 Phenylthiourea (PTU) solution to inhibit pigment formation (Westerfield, 2007). A *TgBAC(pax7a:eGFP)t32239Tg* transgenic line in a *pfeffer* mutant background (herein referred to as pax7a:eGFP) was used for visualizing muSCs *in vivo* (Mahalwar et al., 2014; Knappe et al., 2015).

### Treatment of Larvae With Chemicals

Evans Blue staining to detect damaged muscle was performed by submerging larvae in 0.1% w/v Evans blue diluted in E3 PTU for 1 h in the dark at room temperature (RT) then washed in E3 as previously described (Eliceiri et al., 2011; Smith et al., 2015).

Bromodeoxyuridine (BrdU) labeling was performed by incubating larvae in a solution of 10 mM BrdU (Sigma) diluted in E3 for 24 h.

DAPT (Sigma) was reconstituted to a stock concentration of 40 mM in fresh DMSO, aliquoted and stored at −20°C. The stock solution was then serially diluted in DMSO to 10 mM, and then to a 100 μM working concentration in E3 PTU media as previously described (Dyer et al., 2014). Treated larvae were incubated at 25.8°C in the dark for 24 h.

### Needlestick Injury

Larvae were anesthetized in 0.004% w/v Tricaine (Sigma-200 mg/ml) in E3 media and immobilized in 1.5% w/v low melt agarose (Sigma) (Snider and Clegg, 1975). Larvae were orientated to expose the left lateral muscle. Using a sharpened tungsten wire mounted in a micromanipulator, larvae were injured in the 13th left ventral myotome. Injured larvae were then carefully removed from the agarose and placed in multi-well dishes containing fresh E3 PTU (Knappe et al., 2015).

### Immunolabeling

Larvae were euthanized with 0.4% w/v Tricaine and fixed in 4% w/v paraformaldehyde (PFA) overnight at 4°C. Larvae were washed in 0.1% PBT (1× PBS, 0.1% v/v Tween-20), followed by a methanol series (25, 50, and 75% v/v methanol in distilled H_2_O for 5 min), moved to 100% methanol at −20°C overnight. Fixed larvae were put through a reverse methanol series (75, 50 and 25% v/v in 0.1% v/v PBT), followed by permeabilization in 10 μg/mL proteinase K (3 dpf for 30 min and 7 dpf for 70 min). Once the excess proteinase K had been rinsed away with 0.1% v/v PBT, samples were blocked in 10% v/v newborn calf serum (NBCS) for 1 h at room temperature and incubated with primary antibody overnight at 4°C. For detection of BrdU, samples were treated with 2 M HCl for 1 h at room temperature. HCl was subsequently neutralized by washing in 0.1 M borate buffer (0.62 g Boric acid, 1.5 ml 75 mM NaCl in 100 ml water; pH 8.5), then washed with PBT containing 1% v/v Triton-X and 1% v/v DMSO. Primary antibodies used included mouse anti-Pax7 (developed by A. Kawakami at the Tokyo Institute of Technology, obtained from the Developmental Studies Hybridoma Bank, created by the NICHD of the NIH and maintained at The University of Iowa, Department of Biology, Iowa City, IA, United States), chick anti-GFP (AB16901 Millipore), rat anti-BrdU (ab6326 Abcam), rabbit anti-Myogenin primary antibody (m-2250 sc-576 Santa Cruz Biotechnology). Samples were then washed in 0.1% PBT, blocked in 10% v/v NBCS or 5% v/v goat serum for 1 h at room temperature and incubated with Alexa conjugated secondary antibodies (Invitrogen) at 4°C overnight. Finally, samples were washed in 0.1% PBT, post-fixed in 4% w/v PFA for 30 min at room temperature prior to addition of DAPI or Hoechst-33342.

### Imaging

For live imaging, larvae were anesthetized in 0.004% w/v Tricaine and immobilized in 1.5% w/v low melt agarose in E3 on a glass bottomed dish with size 0 glass (IBL). Time-lapsed images were acquired on a 7MP multiphoton microscope (Carl Zeiss) using a ×20 water dipping objective (NA = 1). Z-stacks encompassing the total myotome (with 1 μm Z-slices) were captured every 10 min from 1 to 16.83 h post-injury (hpi). Larvae processed by immunolabeling were scanned on a Leica SP5 microscope using a ×20 air objective (NA = 0.7). Z-stacks (0.99 μm) were captured at a resolution of 512 × 512 pixels and each channel was averaged three times.

### Image Processing and Cell Counting

Images were processed using Fiji (Schindelin et al., 2012). Brightness and contrast were adjusted to identify eGFP, BrdU, or Myog expressing cells and the “remove outlier” function utilized to remove non-specific signals by selecting bright spots which have a radius 2–4 pixels at a threshold of 10–50. Images of larvae labeled by Myog were processed by removing the background using the image calculator function “AND” to identify Hoescht+ nuclei with Myog labeling. Cells were manually counted from z-stacks within the myotome or vertical myosepta. Images from time-lapsed movies were corrected for drift using the “Correct 3D Drift” function (Parslow et al., 2014).

### Injury Size Calculations

Volumetric quantification of injured muscle labeled with Evans Blue was conducted using Fiji. Brightness and contrast were adjusted to visualize the injured region and remove non-specific background signals. The threshold and measure function on Fiji were used to measure the area labeled by Evans Blue for each Z-slice. The area for each slice was then used to calculate a slice volume (this is equivalent to the area as each Z–interval = 1 μm) and the total volume of the injury calculated from their summed values. In order to calculate the volume of the entire myotome a transmitted image of the myotome at the lateral and medial extent were acquired and their area calculated (V1, V2). Intermediate areas of the myotome at each Z level were extrapolated from V1 and V2 and used to generate a volumetric measure of the total myotome.

### Statistics

Statistical analysis was conducted using R (v4.0.2) running in R-studio (v1.1.463; RStudio Team, 2016). The Supplementary Methods Section details the packages used, and the R function used for each calculation is given in brackets (R:).

Statistical tests were selected based on normality and scedasticity of the data. Normality was assessed by the Shapiro–Wilk test and scedasticity using the Bartlett test or Fligner-Killeen test for parametric and non-parametric data, respectively.

Multi-parametric analysis was conducted by analysis of variance (ANOVA). Data which is non-parametric were first aligned, transformed and ranked using the ARTool package (Wobbrock et al., 2011) prior to the ANOVA (Tables 1, 2 and Supplementary Tables 1, 2; see Supplementary Methods for details). To account for multiple comparisons pairwise comparisons were performed by 1-way ANOVA followed by Tukey’s *post hoc* tests for parametric data. Non-parametric pairwise comparisons were performed using Kruskal-Wallis tests with Dunn’s *post hoc* tests and Benjamini-Hochberg correction.

**TABLE 1:** Results of 2-way ANOVA tests for significant effects of age and injury on the number of muSCs in the myotome.

2-way ANOVA	
Variable	*p*-value
Age	0.0022
Injury	3.61e-06
Age: Injury	0.2745

*2-way ANOVA was performed to test whether there were significant differences in the number of pax7a:eGFP-expressing cells in the myotome of uninjured and injured zebrafish larvae at 4 and 8 dpf. The effects of age, injury, and interactions are shown with *p*-values indicating significance.*

**TABLE 2:** Results of 3-way ANOVA tests for significant effects of age, injury, and DAPT on muSC number.

3-way ANOVA	
Variable	*p*-value
Injury	4.2870e-07
Age	0.5154011
DAPT	2.0126e-08
Injury: Age	0.0059868
Injury: DAPT	5.2814e-07
Age: DAPT	0.3246023
Injury: Age: DAPT	0.5171759

*3-way ANOVA was performed to test whether there were significant differences in the number of pax7a:eGFP-expressing cells in the myotome of uninjured and injured zebrafish larvae treated with DMSO or DAPT at 4 and 8 dpf. The effects of age, injury, drug, and interactions are shown with *p*-values indicating significance.*

Power was calculated using paired tests. Parametric data was analyzed by Student’s *t*-test and non-parametric data analyzed by Wilcoxon-Mann-Whitney u rank sum (WMW) test (Tables 3–5). The alpha-level was set to 0.05 and ideal sample size at 80% power was calculated. The effect size was determined using Cohen’s d.

**TABLE 3:** Calculation of statistical power for comparing the number of muSCs in 4 and 8 dpf zebrafish larvae after muscle injury.

Comparison	Statistical test	95% CI	Effect size	*p*-value	Power (%)	Ideal sample size (n)
4 dpf uninjured	Student’s *t*-test	3.91, 7.09	3.63	0.00009	99.98	3
4 dpf injured		9.72, 13.62				
8 dpf uninjured	Student’s *t*-test	3.43, 13.24	2.36	0.0022	95.64	5
8 dpf injured		14.6, 19.8				
4 dpf uninjured	Student’s *t*-test	3.91, 7.09	0.82	0.19	24.86	25
8 dpf uninjured		3.43, 13.24				
4 dpf injured	Student’s *t*-test	9.71, 13.62	2.51	0.0015	97.35	4
8 dpf injured		14.6, 19.8				

*Power analysis comparing cell numbers obtained from uninjured and injured zebrafish larvae at 4 and 8 dpf. Data is parametric and homoscedastic, therefore a Student’s *t*-test was used to calculate the 95% confidence interval (CI), *p*-value, power, and ideal samples size (at 80% power). Effect size was calculated using Cohen’s d. The sample size calculated has been rounded up to the nearest whole number and is per group (N × 0.5).*

**TABLE 4:** Calculation of statistical power for comparing the number of muSCs after muscle injury in 4 dpf zebrafish larvae in the presence of DAPT.

Comparison	Statistical test	90% CI	Effect size	*p*-value	Power (%)	Ideal sample size (n)
4 dpf uninjured DMSO	WMW test	4, 8	3.43	0.0049	>99.99	3
4 dpf injured DMSO		11, 18				
4 dpf uninjured DAPT	WMW test	6, 9	0.14	0.87	60.00	699
4 dpf injured DAPT		6, 10				
4 dpf uninjured DMSO	WMW test	4, 8	0.83	0.20	39.20	23
4 dpf uninjured DAPT		6, 9				
4 dpf injured DMSO	WMW test	11, 18	2.32	0.015	>99.99	5
4 dpf injured DAPT		6, 10				
4 dpf uninjured DMSO	WMW test	4, 8	0.82	0.25	26.10	29
4 dpf injured DAPT		6, 10				
4 dpf uninjured DAPT	WMW test	6, 9	2.86	0.0062	>99.99	3
4 dpf injured DMSO		11, 18				

*Power analysis comparing cell numbers obtained from uninjured and injured 4 dpf zebrafish larvae which were treated with DMSO or DAPT. Data is non-parametric and homoscedastic, therefore a Wilcoxon Mann Whitney u rank sum test was used to calculate the 90% confidence interval (CI), *p*-value, power, and ideal samples size (at 80% power). Effect size was calculated using Cohen’s d. The sample size calculated has been rounded up to the nearest whole number and is per group (N × 0.5).*

**TABLE 5:** Calculation of statistical power for comparing the number of muSCs after muscle injury in 8 dpf zebrafish larvae in the presence of DAPT.

Comparison	Statistical test	90% CI	Effect size	*p*-value	Power (%)	Ideal sample size (n)
8 dpf uninjured DMSO	WMW test	4, 9	2.30	0.017	>99.99	5
8 dpf injured DMSO		8, 9				
8 dpf uninjured DAPT	WMW test	4, 7	0.51	0.51	5.3	77
8 dpf injured DAPT		5, 8				
8 dpf uninjured DMSO	WMW test	4, 9	0.75	0.27	21.40	26
8 dpf uninjured DAPT		4, 7				
8 dpf injured DMSO	WMW test	8, 25	2.42	0.0081	>99.99	4
8 dpf injured DAPT		5, 8				
8 dpf uninjured DMSO	WMW test	4, 9	0.25	0.71	18.20	181
8 dpf injured DAPT		5, 8				
8 dpf uninjured DAPT	WMW test	4, 7	2.63	0.0049	>99.99	3
8 dpf injured DMSO		8, 25				

*Power analysis comparing cell numbers obtained from uninjured and injured 8 dpf zebrafish larvae which were treated with DMSO or DAPT. Data is non-parametric and homoscedastic, therefore a Wilcoxon Mann Whitney u rank sum test was used to calculate the 90% confidence interval (CI), *p*-value, power, and ideal samples size (at 80% power). Effect size was calculated using Cohen’s d. The sample size calculated has been rounded up to the nearest whole number and is per group (N × 0.5).*

The variability of data around the mean has been expressed by standard deviation (SD). All graphs were produced using GraphPad Prism (version 8.3.0), displaying individual data points and error bars which represent ± SD.

## Results

### Quantification of Needle Stick Injuries Using Evans Blue

**FIGURE 1:**
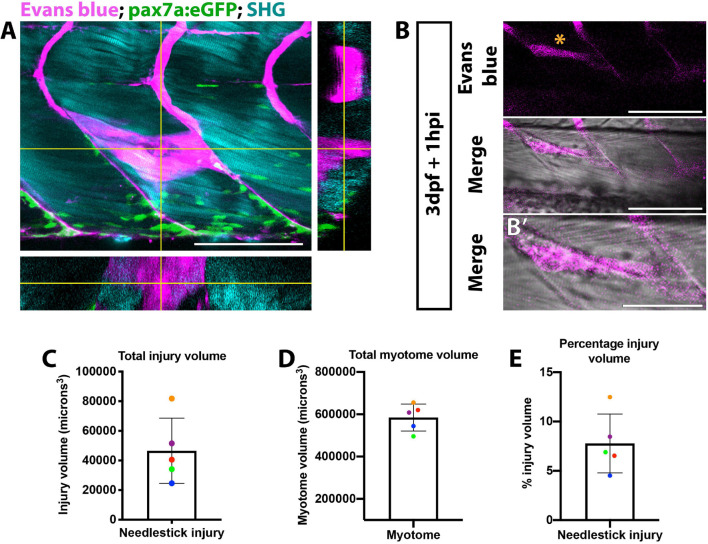
Visualization of muscle injury using Evans blue reveals low variability of injury size caused by needlestick. Evans blue labeling of damaged muscle **(A,B)** was visualized on a multiphoton microscope and localizes with perturbed myofibre morphology **(B’)** at needlestick wounds (asterisk) in 3 dpf larvae at 1 h post-injury. Maximum projection **(A)** with orthogonal sections in planes YZ (right) and XZ (bottom) showing Evans blue staining relative to myofibres (visualized by second harmonic generation; SHG) and pax7a:eGFP expressing muSCs. Volumetric measure of the **(C)** total myotome and **(D)** injured region were calculated by measuring injury size for each section, and the percentage injury size of the total myotome calculated **(E)**. Each larvae (*n* = 5) has been color coded to highlight the variance in myotome and injury size. Error bars display standard deviation. Scale bars: 100 μm **(A,B)**, 50 μm **(B’)**.

In order to enable comparisons of muSC responses to manipulations of key variables we first aimed to establish a highly reproducible method of injury. Standard methods for inducing injury in muscle of zebrafish include injection of myotoxic chemicals such as cardiotoxin (Seger et al., 2011) or application of a sharp needle (Gurevich et al., 2016; Pipalia et al., 2016; Nguyen et al., 2017). Such methods often result in an injury spanning multiple myotomes which elicits a strong muSC response. Although effective, these methods lack precision and reproducibility, making it difficult to determine how muSC responses are dictated by defined variables, hence we used a sharpened needle to cause a precise and controlled injury. To visualize the injury volume we applied Evans blue, which binds to damaged myofibres and fluoresces at far-red wavelengths (Hamer et al., 2002). Evans blue diluted to 0.1% v/v in E3 effectively bound to damaged tissue within 60 min of application and could be seen to co-localize with a second harmonic signal from the polarized myofibres at all depths of the injury (Figure 1A and Supplementary Movie 1). To quantify the injury volume, we measured injury area at each z-interval then used this to calculate an extrapolated volume (Figures 1B,B’). By comparing this relative to the total myotome volume we could estimate the injury extent. Injury size varied between 2.4−8.2×10^4^microns^3^ with a majority (4/5) of injuries in the range between 2.4−5.3×10^4^*microns*^3^ (Figure 1C). To understand whether variability of injury was related to larval size, we also measured the volume of the injured myotome (Figure 1D) and calculated the relative proportion of muscle injured (Figure 1E). We observe that needle stick injury damages approximately 7% of the myotome with low variability (±5%) between animals.

### Responses of Muscle Satellite Cells Are Injury Size Dependent

**FIGURE 2:**
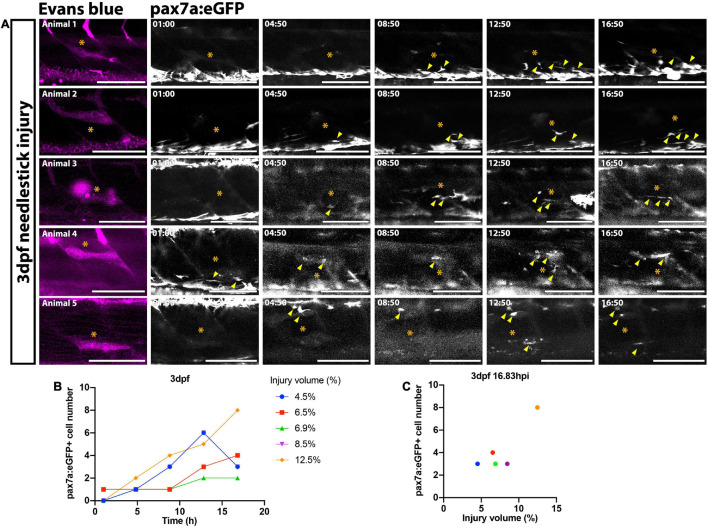
Time-lapsed imaging reveals the extent of the muSC response is related to the injury size. Images from time-lapsed movies reveal pax7a:eGFP-expressing muSCs (arrowheads) respond to injury (asterisk) labeled with Evans Blue (magenta) in the myotome of five representative 3 dpf larvae (**A**, animals 1–5). Images were acquired on a multiphoton microscope and maximum intensity projections generated. The number of pax7a:eGFP^+^ cells in the myotome was quantified and plotted against time **(B)** and injury volume **(C)**. Each colored line **(B)** or dot **(C)** represents a single animal. Scale bars: 100 μm **(A)**.

The extent of the muSC response relative to the extent of tissue damage is likely to be an important factor dictating effectiveness of the regenerative response. To determine how variable the muSC response to tissue damage was in our injury model we imaged muSCs in the myotome of pax7a:eGFP larvae following Evans Blue staining by multiphoton time-lapsed imaging (Supplementary Movie 2). We quantified the number of bright GFP+ muSCs migrating into the myotome and calculated the injury size at defined timepoints relative to onset of injury (Figure 2A). After injury the number of muSCs in the injured myotome increased from  0.4 ± 0.90 to 4.2 ± 2.17 cells over a period of 16.83 h (Figure 2B). Differences in the number of muSCs migrating into the myotome could be seen when comparing between larvae. To determine variability of the muSC response compared to injury size we plotted values obtained at the end of the time-lapse, 16.83 hpi (Figure 2C). There was little variation between animals with injury volume ranging between 4 and 13% with a median of 7% compared to an average of 4.2 muSCs per myotome.

### Expansion of the Resident Muscle Satellite Cell Population Occurs During Larval Development

**FIGURE 3:**
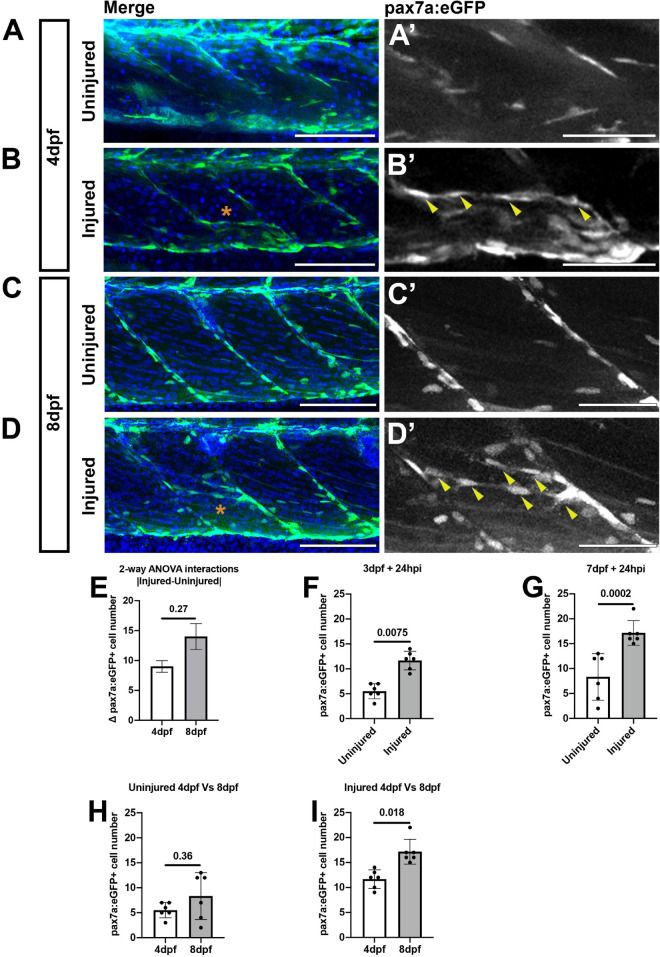
Muscle injury results in an increased number of pax7a:egfp-expressing cells within the myotome at 3 and 7 dpf. Projections of confocal z-stacks of the myotome in uninjured **(A,C)** and injured **(B,D)** 3 or 7 dpf pax7a:eGFP larvae at 24 hpi with nuclei labeled with DAPI. MuSCs (arrowheads) are recruited to the site of injury (asterisk) and align with myofibres at both stages **(A’–D’)**. Quantification of pax7a:egfp-expressing muSCs in uninjured and injured 3 and 7 dpf larvae at 24 hpi. The magnitude of change to the number of muSCs responding to injury (Δpax7a:egfp+) is significantly different between 4 and 8 dpf larvae (*p* > 0.05, **E**). Pairwise comparisons reveals a significant increase in the number of muSCs at both stages as a consequence of injury (*p* < 0.05, **F,G**). There is no significant difference in the number of muSCs between uninjured animals at 4 and 8 dpf **(H)**, but there are more muSCs in injured 8 dpf larvae relative to injured 4 dpf larvae (*p* < 0.05, **I**). Significant differences were tested by 2-way ANOVA (*n* = 47) with Tukey’s HSD *post hoc* test. Error bars represent standard deviation, and values above comparison bars represent significance (*p*-values). Scale bars: 100 μm **(A–D)**, 50 μm **(A’–D’)**.

Many studies investigating tissue regeneration in zebrafish have focused on developmental stages between 3 and 4 dpf. We and others have found extensive changes occur to cell populations in the myotome between 3 and 5 dpf (Hollway et al., 2007; Roy et al., 2017) suggesting that injury may affect developmental programs at these stages. To determine if larval stage affects our ability to discriminate changes to muSC responses to injury we therefore compared the number of muSCs in the myotome of 3 and 7 dpf larvae at 24 hpi by confocal microscopy (Figures 3A–D). Imaging of the myotome with confocal microscope allowed us to detect all GFP+ muSCs, including those not easily detectable by multiphoton microscopy due to their dim fluorescence. In uninjured control larvae, the majority of pax7:eGFP+ cells reside at the myosepta of the myotome at both 4 (Figures 3A,A’) and 8 dpf (Figures 3C,C’). At both stages there were few pax7a:eGFP+ cells within the myotome (5.5 ± 1.52 at 4 dpf and 8.33 ± 4.68 at 8 dpf). At 24 hpi injury, pax7a:eGFP+ cells were observed at the site of injury and were aligned with adjacent myofibres in both 4 (Figures 3B,B’) and 8 dpf larvae (Figures 3D,D’).

To test whether the number of muSCs in a myotome changes during development we counted pax7a:eGFP+ cells in the myotome of injured and uninjured control larvae. To test for the importance of age and injury for affecting muSC number we applied a 2-way ANOVA. Although, both variables were significantly associated with changes to muSC number (*p* < 0.05; Table 1) there is no interaction effect between age and the injury response (Table 1 and Figure 3E). This may be due to differences in myotome size between 4 and 8 dpf larvae. The myotome grows by addition of new myofibres and growth of myofibres at larval stages (Hinits et al., 2011). As injuries performed on animals at 3 and 7 dpf are of comparable size the relative injury size is smaller in older animals and so may not promote the same degree of response by the muSC population. To understand how either age or injury affected muSC number we performed *post hoc* tests. We observed an increased number of muSCs in the myotome following injury at both 4 (Figure 3F) and 8 dpf (Figure 3G). We found that there was no difference in the number of muSCs between larval age in an absence of injury (4 dpf: 5.5 ± 1.52, 8 dpf 8.33 ± 4.68; *p* > 0.05; Figure 3H). We also found that there was a significant difference following injury (*p* < 0.05, Figure 3I) between developmental stages with more muSCs present in older larvae (17.17 ± 2.48) compared to younger larvae (11.67 ± 1.86) despite the relative injury being smaller in older animals.

### Notch Regulates Muscle Satellite Cell Responses to Injury in Zebrafish Larvae

Notch signaling is a critical regulator of muSC quiescence and proliferation (Bjornson et al., 2012; Mourikis et al., 2012). It is not known if Notch has a conserved role in regulating muSCs in zebrafish during regeneration. We therefore investigated the requirement by muSCs for Notch activity by inhibiting Notch activation with the well characterized pharmacological inhibitor of γ-secretase, DAPT (Geling et al., 2002).

To test the prediction that an absence of Notch activity would result in fewer muSCs expressing Pax7+ we quantified the number of Pax7+ cells in the myotome of injured 3 dpf larvae in the presence or absence of DAPT. We observed fewer Pax7+ myoblasts within the myotome of animals treated with 100 μM DAPT compared to control animals (*p* < 0.05) at 24 hpi. Interestingly we also noted a reduction of Pax7+ cells at the vertical myoseptum (VM) in animals exposed to DAPT (Supplementary Figure 1).

**FIGURE 4:**
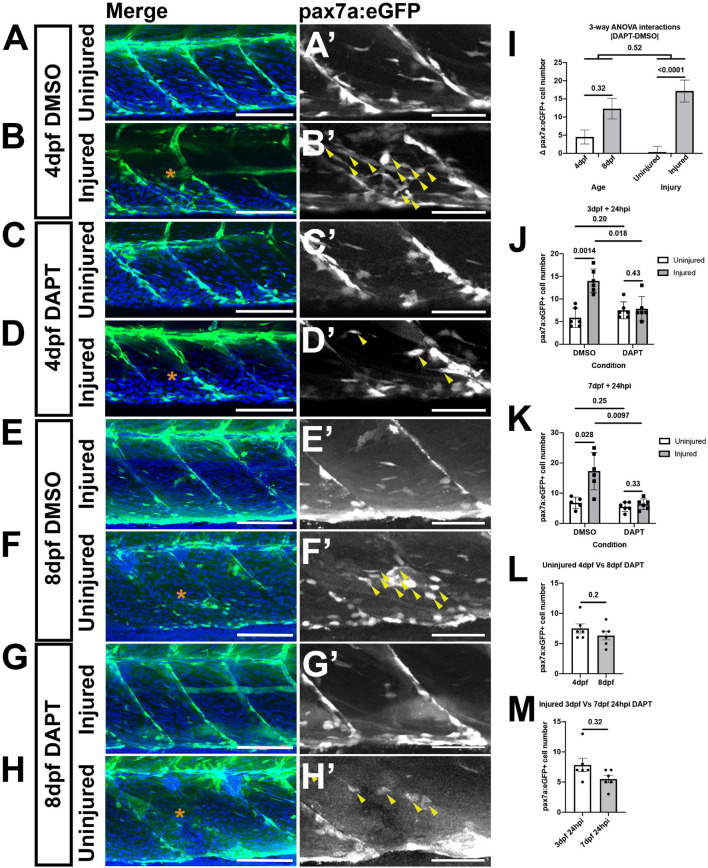
A loss of Notch activity attenuates the muSC response to injury. Projections of confocal stacks of the myotome in uninjured **(A,C,E,G)** and injured **(B,D,F,H)** pax7a:eGFP larvae at 3 dpf **(A–D)** or 7 dpf **(E–H)**. Animals were treated with 1% v/v DMSO **(A,B,E,F)** or 100 μM DAPT **(C,D,G,H)** prior to and after injury, fixed at 24 hpi and nuclei labeled with DAPI. There are more muSCs expressing eGFP (arrowheads) recruited to the injury (asterisk) in DMSO treated larvae **(B’,F’)** compared to DAPT treated larvae **(D’,H’)**. Quantification of pax7a:egfp-expressing muSCs in uninjured and injured 3 and 7 dpf larvae treated with DMSO or DAPT. Tests for significant differences in muSC number due to injury, DAPT or age revealed that injury affected the number of muSCs present (*p* < 0.05), but DAPT treatment nor developmental stage did (*p* > 0.05, **I**). Pairwise comparisons revealed that injury-induced changes to the number of muSCs is attenuated by treatment with DAPT (*p* < 0.05, **J,K**). There is no significant difference in the number of muSCs in the myotome of uninjured 4 and 8 dpf larvae treated with DAPT (*p* > 0.05, **L**). Likewise, there is no difference of muSC number in the myotome of injured 4 and 8 dpf larvae treated with DAPT (*p* > 0.05, **M**). Significant differences were tested by 3-way ANOVA following transformation by ART (*n* = 47) and *post hoc* tests performed using a Dunn’s test with Benjamini and Hochberg correction. Error bars represent standard deviation, and values above comparison bars represent significance (*p*-values). Scale bars: 100 μm **(A–H)**, 50 μm **(A’–H’)**.

To determine whether similar reductions of pax7a:egfp-expressing muSCs occurred in response to DAPT larvae at 3 and 7 dpf were treated with 100 μM DAPT (Figures 4A–H). A 3-way ANOVA was used to test for differences in muSC number due to age, injury or DAPT treatment. Injury and DAPT treatment significantly contributed to changes to muSC number but age did not (*p* < 0.05, Table 2). Again, this may reflect differences in the relative injury size and therefore obscure any age-dependent differences. Although we had identified a significant 2-way interaction between injury and DAPT there was no difference in the response to DAPT due to developmental stage (Table 2 and Figure 4I). This reveals that Notch inhibition has similar effect on the muSC response to injury at both 3 and 7 dpf.

To understand how DAPT affected muSC number relative to age or injury we performed *post hoc* tests. DAPT treatment results in no changes to the number and localization of muSCs in the myotome of uninjured larvae compared to DMSO treated control larvae at 4 dpf (*p* > 0.05, Figures 4A’,C’,J) and 8 dpf (Figures 4E,G’,K). In contrast, there were fewer muSCs within the myotome following injury when animals were treated with DAPT compared to DMSO at both 4 dpf (*p* < 0.05, Figures 4B’,C’,J) and 8 dpf (*p* < 0.05, Figures 4F’,H’,K).

To understand if DAPT treatment affected muSC number in the myotome differently due to developmental stage we compared uninjured (Figure 4L) and injured (Figure 4M) animals treated with DAPT but did not see any significant differences (*p* > 0.05). This reveals that DAPT treatment does not affect the number muSCs within the myotome but loss of Notch signaling prevents the expansion of the muSC population in response to injury at both 3 and 7 dpf.

To confirm that DAPT is acting to specifically inhibit Notch activity we evaluated the consequences of over-expressing a dominant negative version of the Suppressor of Hairless gene (dnSu(H)), orthologous to the mammalian transcription factor RBP-J (Latimer et al., 2005), when muSCs were responding to injury. A global over-expression of dnSu(H) was performed by heat-shock induction of the HS:dnSu(H) transgene in pax7a:egfp larvae, animals were injured then fixed after 24 h. Over-expression of dnSu(H) did not affect the number of GFP+ cells in the myotome of uninjured animals (*p* > 0.05). In injured animals there was a highly significant difference in the number of GFP+ muSCs between wildtype siblings and animals expressing dnSu(H). Animals over-expressing dnSu(H) had far fewer muSCs, comparable to uninjured animals and similar to results from treating animals with DAPT (Supplementary Figure 2 and Supplementary Table 4).

### Notch Inhibition Reduces Muscle Satellite Cell Proliferation in Response to Injury

**FIGURE 5:**
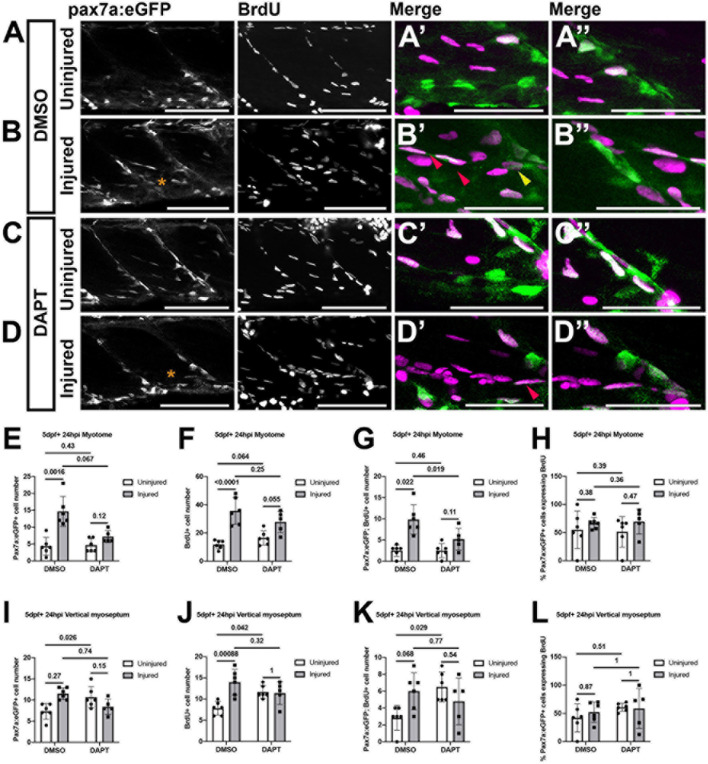
Proliferation of muSCs during regeneration and homeostasis is Notch dependent. Projections of confocal stacks **(A–D)** of the myotome **(A’–D’)** and vertical myoseptum **(A”–D”)** in uninjured **(A,C)** and injured **(B,D)** 5 dpf pax7a:eGFP larvae incubated with BrdU for 24 h. Larvae were treated with 1% v/v DMSO **(A,B)** or 100 μM DAPT **(C,D)** after injury and detection of BrdU and eGFP was performed by immunolabeling. MuSCs with (red arrowheads) and without BrdU labeling (yellow arrowheads) are recruited to the injury (asterisk) site **(B’,D’)**. The number of cells expressing pax7a:eGFP **(E,I)**, incorporating BrdU **(F,J)** or both **(G,K)** were counted in the myotome **(E–G)** and both vertical myoseptum **(I–K)** of animals treated with DMSO or DAPT. The proportion of pax7a:eGFP-expressing muSCs incorporating BrdU was calculated for both myotome **(H)** and vertical myoseptum **(L)**. There were significantly fewer GFP+ muSCs incorporating BrdU after injury in the presence of DAPT compared to DMSO treated control animals (*p* < 0.05; **G**). There is no significant change to the number of GFP+ muSCs incorporating BrdU at the vertical myoseptum of injured animals in the presence of DAPT (*p* > 0.05; **K**). In contrast, although there are only few cells present, there is a significant increase in the number of muSCs with BrdU labeling at the myoseptum in uninjured animals in the presence of DAPT (*p* < 0.05; **K**). Significant differences were tested by 2-way ANOVA (*n* = 23) with Tukey’s HSD *post hoc* test or by transforming data by ART and performing 2-way ANOVA followed by a Dunn’s test with Benjamini and Hochberg correction. Error bars display standard deviation, and values above comparison bars indicate significance (*p*-values). Scale bars: 100 μm **(A–D)**, 50 μm **(A’–D’,A’–D”)**.

The failure of the muSC population to expand in response to injury in the presence of DAPT could reflect a requirement for Notch in regulating proliferation as has been described in mammals (Brack et al., 2008). To test whether proliferation of muSCs is impaired by DAPT treatment, we measured BrdU incorporation by pax7a:eGFP expressing muSCs in the myotome of 8 dpf larvae (Figure 5). A 2-way ANOVA was used to test whether injury or DAPT treatment altered the number of proliferating muSCs. Injury causes a significant increase in the number of proliferating muSCs as we have described previously (Knappe et al., 2015). In contrast, DAPT treatment significantly reduced the number and proportion of muSCs incorporating BrdU. There was also a significant interaction effect between injury and DAPT treatment, suggesting that DAPT alters the muSC injury response (*p* < 0.05, Supplementary Table 2). There are very few proliferating muSCs in uninjured larvae treated with DMSO (2.50 ± 1.38; Figure 5A’) or DAPT (2.50 ± 1.64; Figure 5C’). In injured larvae there was an increased number of proliferating muSCs compared to uninjured larvae in animals treated with either DMSO (Figure 5B’) and DAPT (Figure 5D’). There was a significant reduction in the number of proliferating muSCs in injured larvae treated with DAPT (5.20 ± 2.59) compared to those treated with DMSO (9.83 ± 3.49; *p* < 0.05, Figure 5G). However, this did not reflect a change to the relative proportion of proliferating pax7a:eGFP-expressing cells within the myotome (Figure 5H), but rather is due to a decrease in the overall number of muSCs present (Figure 5E).

Descriptions of Pax3-expressing muSCs in zebrafish have highlighted the vertical and horizontal myosepta as a potential site for a stem cell niche for muSCs (Nguyen et al., 2017). The VM is enriched for molecules associated with the ECM and cell adhesion including Fibronectin and Laminin (Goody et al., 2012). Our characterization of muSC responses to injury has shown that pax7a-expressing cells detach from the VM and migrate toward damaged myofibres (Knappe et al., 2015). To understand if Notch regulates muSCs within this potential niche we evaluated the effect of Notch inhibition on pax7a:eGFP-expressing muSCs located at the VM (Figures 5I–L). 2-way ANOVA tests showed that DAPT treatment affected the number of pax7a:eGFP+ muSCs at the VM relative to injury (interaction effect; Supplementary Table 2). By conducting pairwise analysis, we found that there are very few proliferating muSCs within the VM of uninjured animals treated with DMSO (2.83 ± 1.47; Figure 5A”). This increased following injury, but this was not significant (6 ± 2.19; Figures 5B”,K). In the presence of DAPT, there was a significant increase in the number of proliferating muSCs in the myoseptum of uninjured (6.50 ± 1.76; Figure 5C”) compared to DMSO treated animals (*p* < 0.05, Figures 5A”,K). There was no significant difference of muSC proliferation at the myosepta in injured animals in the presence of DAPT (Figure 5C”) compared to control animals (*p* > 0.05, Figures 5B”,K). This indicates that inhibition of Notch promotes proliferation of muSCs in their niche at the VM in an absence of injury. In contrast an inhibition of Notch lead to a reduced proliferation of muSCs in the myotome as they respond to injury.

The horizontal myoseptum (HM) has been described as a low-cycling niche of pax3a-expressing progenitor cells that contribute to cells resident at the VM at 3 dpf (Nguyen et al., 2017). We therefore examined the HM for changes due to injury and DAPT treatment (data not shown). Only a few pax7a:egfp-expressing cells with BrdU incorporation were detected at the HM in uninjured control animals (2 ± 1.09). This was not significantly different to animals exposed to DAPT (1.83 ± 0.75) or injured animals without DAPT (1.67 ± 1.37) or in the presence of DAPT (1.4 ± 1.52). The low number of cells detected and their presumably long cell cycle resulting in slow incorporation of BrdU, makes it difficult to draw definitive conclusions from this result as to whether resident muSCs at the HM are affected by a loss of Notch signaling.

### Notch Inhibition Leads in an Increase in the Proportion of Differentiating Muscle Satellite Cell in Response to Injury

**FIGURE 6:**
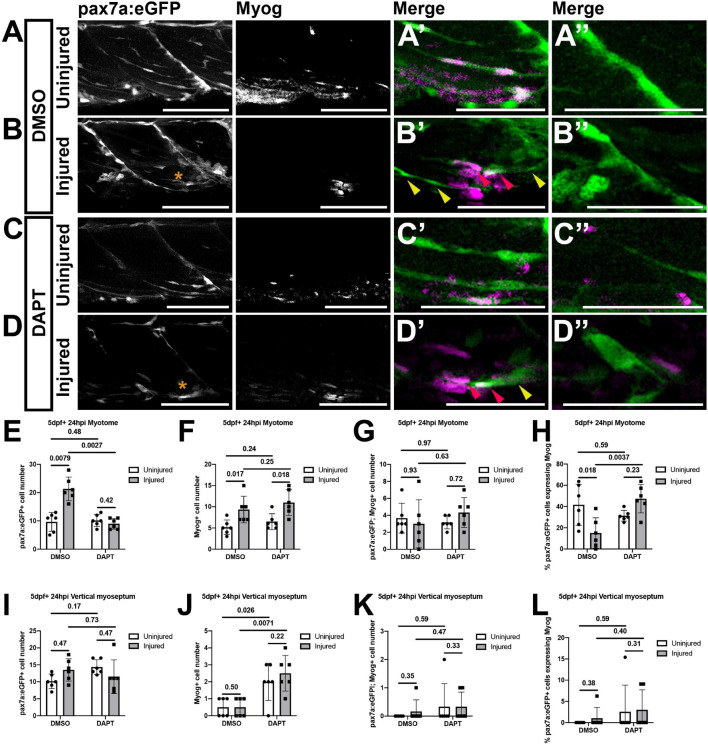
Inhibition of Notch signaling results in an increased differentiation of muSCs responding to injury. Projections of confocal stacks **(A–D)** of the myotome **(A’–D’)** and vertical myoseptum **(A”–D”)** of 5 dpf pax7a:eGFP larvae treated with 1% DMSO **(A,B)** or 100 μM DAPT **(C,D)** prior to **(A,C)** and after injury **(B,D)**, fixed at 24 hpi and labeled with anti-Myogenin. pax7a:GFP expressing muSCs with (red arrowheads) and without Myogenin labeling (yellow arrowheads) are recruited to the injury (asterisk) site **(B’,D’)**. Quantification of pax7a:egfp expressing muSCs **(E,I)**, Myogenin expressing cells **(F,J)** and co-labeled cells **(G,K)** in the myotome **(G,H)** and both vertical myoseptum **(I–K)** reveals no significant difference in the number of muSCs expressing Myogenin in DAPT treated compared to DMSO treated animals (*p* > 0.05, **G,K**). Examination of the proportion of pax7a:eGFP expressing muSCs expressing Myogenin in the myotome **(H)** and vertical myoseptum **(L)** reveals a significantly increase in those expressing Myogenin in the presence of DAPT following injury in the myotome (*p* < 0.05, **H**). Significant differences were tested by 2-way ANOVA (*n* = 24) with Tukey’s HSD *post hoc* test or by transforming data by ART and performing 2-way ANOVA followed by a Dunn’s test with Benjamini and Hochberg correction. Errors bars display standard deviation and values above bars indicate significance (*p*-values). Scale bars: 100 μm **(A–D)**, 50 μm **(A’–D’,A”–D”)**.

In mice knockdown of Notch in muSCs results in them differentiating in a premature S-phase independent manner following muscle injury (Bjornson et al., 2012). To examine if Notch inhibition leads to premature differentiation of muSCs in the zebrafish, we assessed the number of Myogenin (Myog) expressing muSCs in the myotome of larvae treated with DAPT (24 hpi; Figure 6). Using a 2-way ANOVA test we found that injury and DAPT treatment did not alter the number of muSCs expressing Myog. However, when we tested for changes to the proportion of muSCs expressing Myog we observed that there was a significant interaction effect between injury and DAPT (*p* < 0.05, Supplementary Table 3).

There were few Myog expressing muSCs within the myotome of uninjured larvae (3.67 ± 1.75; Figure 6A’) and this did not change following Notch inhibition (3.17 ± 0.75; Figures 6C’,G). Following injury, there was an increase in the number of total Myog expressing cells relative to uninjured controls (Figures 6A’,B’,F). However, there was no change to the number of muSCs expressing Myog in response to injury (Figure 6G). Inhibition of Notch does not alter the number of muSCs expressing Myog after injury (DMSO: 3 ± 2.83, DAPT: 4.33 ± 1.75; Figures 6B’,D’,G). We noted that there are fewer pax7a:eGFP expressing muSCs in the myotome of injured animals when Notch is inhibited (Figure 5E) and found that the proportion of pax7a:eGFP cells expressing Myog significantly increases in the absence of Notch signaling (*p* < 0.05, DMSO: 15 ± 14.38%, DAPT: 47.35 ± 13.35%, Figure 6H). This reveals that inhibition of Notch both reduces the number of proliferating muSCs and results in a higher proportion of muSCs undergoing differentiation in the context of injury.

In the absence of injury, we found that Notch inhibition results in an increased number of proliferating muSCs at the VM. To determine if Notch inhibition leads to a premature differentiation of muSCs within their niche we quantified the number of muSCs expressing Myog at the VM of uninjured animals in the presence of DAPT. We could not detect pax7a:egfp-expressing cells with myog at the VM of uninjured animals and this did not change in the presence of DAPT (Figures 6A”,C”,K). There was an increase in the total number of Myog expressing cells at the VM in response to DAPT treatment (*p* < 0.05, DMSO: 0.5 ± 0.55, DAPT: 2 ± 1.1, Figure 6J). This increased number of Myog-expressing cells resulting from DAPT treatment was not different between uninjured (Figure 6C”) and injured (Figure 6D”) animals. We note there was no change to the number (Figure 6K) or proportion (Figure 6L) of pax7a:eGFP-expressing muSCs expressing Myog in response to DAPT treatment. This suggests there are other myogenic cells within the VM that undergo a premature differentiation in the absence of Notch activity in an injury-independent manner. There were very few cells showing myog immunoreactivity at the HM in uninjured (0.33 ± 0.52) or injured (0.83 ± 1.60) animals and this did not change significantly in the presence of DAPT (data not shown).

### Muscle Satellite Cells Show Significant Responses to Injury and Notch Inhibition but Not as a Consequence of Larval Stage

Our results showed no significant changes to muSC proliferation in response to DAPT treatment in uninjured animals. However, it is possible that we were not able to identify changes due to the low number of cells present relative to injured animals. In experiments investigating muSC biology in the mouse many hundreds of cells are measured whereas in the myotome of larval stage zebrafish there are far fewer cells. This reflects a potential caveat for inferring statistically significant differences between conditions and highlights the need to develop a robust statistical methodology for determining whether muSC are affected by a specific variable. We therefore determined how variables developmental stage and inhibition of Notch were related to power and effect size (Supplementary Table 1).

Power is influenced by multiple factors, including the variability of the data, effect size, alpha (α) level, and sample size. A comparison of data generated from animals of different stages that were injured or uninjured showed a normal distribution with equal variance. In contrast, data obtained from animals exposed to DMSO or DAPT showed a non-normal distribution with equal variance (Tables 3–5 and Supplementary Figure 1). The effect size represents the difference between two samples means relative to the variance (or spread) of the data (Cohen, 1992; Browne, 2010). We used the data generated from our characterization of muSC at different ages and from injured and uninjured animals to identify a sample size which achieves a power of 80% at an alpha level of 0.05. Confidence intervals (CI), the probability of making a type I error (rejecting the null hypothesis that two means are equal when this is true, represented by the *p*-value), power and effect size were calculated (Tables 3–5). This revealed that datasets comparing injured to uninjured animals showed high power (96–99%; Table 3), in agreement with the low *p*-value, non-overlapping CI and large effect size. This was equally true for both larval stages, in which a sample size of *n* = 3 (4 dpf) and *n* = 5 (8 dpf) were required to detect a significant difference due to injury with high power. In contrast, a power analysis of datasets generated from uninjured animals revealed low power (25%) when testing for differences due to larval stage. The sample size required to achieve an effect size of 0.8 at 80% power was *n* = 25 when comparing 4 and 8 dpf uninjured larvae. The low power achieved when comparing these datasets and the need to use many samples to discriminate differences between these stages suggests there is either considerable heterogeneity between samples or little difference in the number of muSCs between larval stages. It is likely that the difference in relative injury size compared to the myotome size increasing due to growth between 3 and 7 dpf further reduced power. We then evaluated the sample size needed for identifying differences due to larval stage in the presence of an injury. Using a sample size of *n* = 4 showed an effect size >2.5 with high power (97%), indicative of a large difference in muSC responses to injury between developmental stages.

We evaluated power and effect size for datasets in which we had inhibited Notch activity using DAPT (Tables 4, 5). In uninjured larvae we observed an effect size >0.8 with 39% power when comparing uninjured 4 dpf larvae treated with DAPT compared to control (DMSO treated) larvae, and an effect size >0.75 with power 21% power when comparing uninjured 8 dpf larvae. The ideal sample size for identifying differences between uninjured larvae treated with DAPT compared to control larvae is *n* = 23 and *n* = 26 at 4 and 8 dpf, respectively. In contrast, in injured larvae, we identified an effect size >2.3 and >99% power when comparing 4 dpf larvae treated with DAPT to control animals and an effect size >2.4 with power >99% power when comparing 8 dpf animals. We can therefore conclude that the ideal sample size for identifying differences of the number of muSCs in DAPT treated compared to control animals in the presence of an injury is *n* = 5 and *n* = 4 at 4 and 8 dpf, respectively.

## Discussion

In this work we reveal that Notch signaling is required both for proliferation of muSCs in response to injury and to regulate muSCs in their putative niche at the myosepta. This is analogous to the described roles of Notch in maintaining quiescence of muSCs and promoting proliferation in response to injury. We further demonstrate that Notch is required for expansion of the muSC pool in response to injury at both early and later larval stages in zebrafish. Our observation that inhibition of Notch results in muSCs at the VM exhibiting an increased proliferation leads us to suggest that this resembles the activation of muSCs in adult mammals in response to a loss of Notch activity. This implies that regulation of muSCs at the myoseptum is analogous to that of the muSC niche in adult muscle (Brack et al., 2008; Bjornson et al., 2012; Mourikis et al., 2012). Overall our results suggest that Notch has a conserved function in regulating muSCs in both fish and mammals and that larval stage zebrafish are a valid model for investigating this process.

### Notch Has a Conserved Role in Regulating Muscle Satellite Cell Proliferation

Dissecting the molecular and biophysical mechanisms regulating endogenous muscle stem cell function is of critical importance for designing interventions for enhancing lifelong health. A failure to maintain muscle health and strength is a key hallmark of aging and corresponds with a reduced number of muSCs that show a diminished response to tissue damage. The Notch pathway is a major regulator of muSC quiescence and plays an important role in renewal of the resident muSC population (Bjornson et al., 2012; Mourikis et al., 2012; Baghdadi and Tajbakhsh, 2018). Depletion of the Notch target genes Hey1/HeyL or of Notch 1/Notch2 in muSCs results in a break from quiescence in an absence of injury and impaired proliferation (Fujimaki et al., 2018; Noguchi et al., 2019).

A role for Notch in regulating muscle progenitor cell responses to injury has not been previously described in zebrafish. Inhibition of Notch signaling at earlier developmental stages perturbs somitogenesis and results in axial deformities due to disruption of the somite clock (Oates and Ho, 2002). Myofibres show abnormal morphology and muscle formation is impaired in animals mutant for the segmentation clock genes *her1* and *her7* (Lleras-Forero et al., 2020). Abrogation of Notch signaling using DAPT at early larval stages (24 hpf) results in thin and malformed myofibres in the fin as well as a reduced number of Pax7+ progenitor cells (Pascoal et al., 2013). Notch signaling therefore appears to be important for development of muscle in zebrafish, but it is not clear if it also plays a later role in regulating homeostasis as described in mammals.

In amniotes Notch signaling to muSCs is required to maintain quiescence. In mice in which the Notch activated transcription factor RBP-J is ablated at embryonic stages there is a deficit of Pax7+ muscle progenitor cells, which differentiate prematurely (Vasyutina et al., 2007). Similarly, inhibition of Notch in developing chicken embryos results in a deficit of Pax7+ myogenic progenitor cells in the myotome (Picard and Marcelle, 2013; Esteves de Lima et al., 2016). Notch is also required for self-renewal of activated muSCs which will otherwise prematurely differentiate in an absence of Notch-Delta signaling (Yartseva et al., 2020). Our results from larval stage zebrafish reveal that Notch is required for preventing proliferation of muSCs in an absence of injury at larval stages. Inhibition of Notch by application of DAPT results in elevated proliferation of pax7a-expressing muSCs at the VM in an absence of injury. In contrast, in injured animals there is no significant change, suggesting the primary function of Notch is to prevent proliferation under homeostatic conditions, similar to the role of Notch in maintaining quiescence of muSCs in adult muscle of mice.

We found that when comparing muSC number in animals treated by DAPT to control animals in an absence of injury there was low power. This may reflect the short time period of exposure to BrdU (24 h) as it may be too limited to identify sufficient cycling cells. More power could be obtained by increasing the number of animals tested and scanning more injured myotomes to enable quantification of more pax7a-expressing cells (∼6–8 at 3–7 dpf). Our power calculations argue that to achieve a power of >80% we would need to use 23–25 animals in each condition. This is in contrast to experiments investigating the effect of Notch inhibition on muSCs in the context of injury, in which high power can be obtained with few animals (*n* = 4–5). As such it is difficult to make definitive statements about the consequences of inhibiting Notch on the putative stem cell niches in the VM and HM.

### Developmental Stage Affects Zebrafish Regenerative Responses

A majority of studies investigating muSC responses to injury in zebrafish have been performed using animals younger than 5 dpf (Seger et al., 2011; Gurevich et al., 2016; Pipalia et al., 2016). Legislation in the United Kingdom and the EU states animals older than 5 dpf are protected animals as the larvae are free feeding (Directive 2010/63/EU, 2010; Home Office, 2014). However, it is not clear that muSC behavior at younger larval stages are comparable to those observed in later larval and juvenile, yet alone adult stages. In mice and chick, differences of muscle progenitor cells at fetal, juvenile and adult stages have been defined based on their behavior and expression of molecular markers (Gros et al., 2005; Lepper et al., 2009).

Characterization of muscle progenitor cells expressing Pax7 and the related transcription factor Pax3 in mice, reveal important differences due to developmental age (Buckingham and Relaix, 2007). These include differing proliferative, fusion and engraftment potential, elevated expression of Notch target genes Hey1 and HeyL and of Tenascin-C in fetal stage Pax7-expressing muSCs (Tierney et al., 2016). At later stages of development, muscle progenitor cells take up residence in their niche beneath the basal lamina of myofibres. This switch of progenitor cells from a fetal to juvenile program occurs at postnatal day 21 and is Notch-dependent in mice (Bröhl et al., 2012). The extracellular matrix surrounding muSCs also changes during embryonic development and this is an autonomous function of the muSC (Tierney et al., 2016). Quiescence of muSC progenitor cells commences at 7 weeks postnatal and correlates with diminished proliferation (Picard and Marcelle, 2013; Gattazzo et al., 2020). It is unclear whether muscle progenitor cells at larval stages are regulated similarly to adult muSCs in zebrafish. Fusion of Pax7+ muSCs at both larval and adult stages requires Myogenin function (Ganassi et al., 2020). Descriptions of pax7a-expressing cells in adult zebrafish reveal similar proliferative responses to injury as described for larval stages (Berberoglu et al., 2017). Our results argue that Notch signaling plays an important role in regulating muSC responses to injury at larval stages, similar to the role described for muSCs in fetal and adult stage mice.

Fan et al. (2012), have argued that developmental programs regulating muscle progenitors show important differences to those employed during regeneration at adult stages. Although it is true that adult stem cells undergo epigenetic changes on entering quiescence, a number of mechanisms do appear to be utilized at both embryonic and adult stages. One innovative approach to identify regulators of myogenesis demonstrated that a number of small molecule modifiers had similar effects on myogenesis of zebrafish embryonic cells and human iPSCs (Xu et al., 2013). In order to understand the limits of using developing stage animals for modeling of stem cells it is therefore important to define similarities and differences between adult and juvenile stages, as well as species specific differences.

Similar to mammals, pax7 and pax3-expressing progenitor cells are specified during somite development in zebrafish and are regionalized to the anterior somite (Hollway et al., 2007). They subsequently become reorganized to the periphery of the forming myotome to form the ECL and continue to express pax7 genes (Hollway et al., 2007; Stellabotte et al., 2007). Pax7+ muscle progenitor cells from the ECL migrate inward to the myotome to contribute to new myofibres in a number of fish species (Devoto et al., 2006). An ongoing contribution of progenitor cells to muscle growth by hyperplasia is a feature of teleosts. A clonal tracing method was used to describe a mosaic hyperplasia in zebrafish in which the authors argued the continuous existence of an ECL to provide new myofibres for growth (Nguyen et al., 2017). Whether this ECL also contributes to regeneration of muscle or is primarily involved in growth is unclear, although it is worth noting that a significant contribution of pax7a:egfp-expressing cells to regeneration of myofibres in adult zebrafish argues for a dual role of these cells in both growth and repair (Berberoglu et al., 2017).

We have previously described an inward migratory behavior of a pax7a-expressing muscle progenitor cell population from the peripheral extremes of the somite in zebrafish between 3.5 and 5.5 dpf (Roy et al., 2017). Similarly, progenitor cells expressing a pax3a:egfp transgene show an inward migratory behavior and contribute to myofiber formation between 1 and 3 dpf (Nguyen et al., 2017). This inward migration and contribution of pax7a-expressing and pax3a-expressing cells to myofibre growth is characteristic of the ECL. Both pax7a and pax3a-expressing cells take up residence at the vertical and horizontal myosepta of the myotome from around 3 dpf. Myosepta are myotendinous junctions anchoring myofibres and are enriched for components associated with cell adhesion including Fibronectin, Laminin, Vinculin, and Paxillin (Goody et al., 2010; Jacob et al., 2017). An analysis of proliferation in the myotome of 3 dpf larvae by EdU labeling revealed proliferation of pax3a:eGFP cells primarily occurs at the vertical myosepta (Nguyen et al., 2017). However, this is at stages prior to the medial migration of ECL cells and their contribution to new myofibres therefore may be a transient behavior. We find that at later larval stages (7 dpf) proliferation of pax7a-expressing muSCs also occurs primarily at the vertical myosepta, suggesting the VM supports a population of proliferating muSCs at larval stages. We were not able to identify contributions of cells from the HM to regeneration in any of our time-lapsed datasets. Observations of a pax3a:egfp transgenic line suggested that cells migrate from the HM to the VM at 3 dpf (Nguyen et al., 2017). As we found that pax7a:egfp-expressing muSCs at the HM show lower rates of proliferation than at the VM it is possible that the HM could act as a reservoir for muSCs. Long term proliferation assays would be able to clarify this and reveal whether there is a tendency for one anatomical site to contribute to growth as opposed to regeneration. One other important consideration is whether there are discrete populations of muSCs that can contribute preferentially to growth or regeneration. A population of met-expressing muscle progenitor cells was described in the deep myotome of larval staged animals that contribute to regeneration and express pax7 genes (Gurevich et al., 2016). This population of cells persists into adult stages and may be analogous to muSCs in mammals which also express c-Met (Webster and Fan, 2013). How this cell population is related to those expressing pax3a or pax7 genes at adult stages will be important for making comparisons to mammalian muSCs. It is an open question as to whether the muscle progenitor cell populations expressing these genes are analogous to fetal muscle progenitor cells in mammals or represent a cell population transitioning to a stem cell state (Keenan and Currie, 2019). Nonetheless, the location of muscle progenitor cell populations with a slow rate of proliferation argues that the VM may act analogously to a stem cell niche.

In contrast to the contributions of pax7a:egfp and pax3a:egfp-expressing cells to myofiber formation between 3 and 5 dpf, we observed little contribution of pax7a-expressing muSCs to growth at 7 dpf (Knappe et al., 2015). We propose therefore that the persistent localization of slowly proliferating pax7a:egfp-expressing cells to the VM, their minimal contribution to new myofibre formation and lack of migration in an absence of injury at 7 dpf suggests that these cells are transiting to a quiescent state. In contrast, the extensive migration of pax7a-expressing cells and their contribution to myofibre formation in an absence of injury at earlier larval stages reflects a specific developmental behavior of this progenitor cell. This migratory behavior and contribution to myogenesis by muSCs between 3 and 5 dpf suggests they are contributing to secondary myogenesis, but at later stages after 7 dpf they are entering quiescence and contribute far less to growth than at earlier stages. Despite this apparent change in cell behavior, we find that these cells require Notch for their proliferation at both 3 and 7 dpf in response to injury. This role of Notch in promoting muSC proliferation is therefore present at early and later developmental stages in zebrafish, similar to amniotes, and may also regulate proliferation at adult stages.

### Non-autonomous Regulators of the Muscle Satellite Cell Response to Injury

Focal injuries of muscle, using a needlestick, have revealed muSC responses differ dependent on the extent of injury (Knappe et al., 2015; Ratnayake et al., 2021). Modeling of innate immune cell responses to injuries of defined size in *Drosophila* and zebrafish revealed a positive correlation between the injury size and number of cells responding (Weavers et al., 2016). Scalar responses of immune cells to injury-induced signaling are seen for recruitment of neutrophils by an H_2_O_2_ gradient after tail transection (Niethammer et al., 2009). We confirmed that our method of injury resulted in a consistent injury size affecting only a small proportion of the myotome. We find the muSC response showed little variability, implying that there was a high reproducibility between our datasets. This is important as any variability would reduce the power of statistical comparisons between animals and conditions. We did not demonstrate a differential response of muSCs to injury, but note that a number of published reports have interchangeably used small or larger injuries when examining muscle regeneration in zebrafish. As it is unclear if there is a scalar response of muSCs to injury, or if there are thresholds of injury extent that induce different muSC (and other cell) responses, it is difficult to make broad comparisons between studies. It is possible that the muSC response corresponds to the relative level of damage signals released by the myofibre, the extent of the immune response, or signals from other cells and tissues as a consequence of the damage (Pillon et al., 2013; Weavers et al., 2016; Uderhardt et al., 2019). Recruitment of leukocytes to injured tail fins is induced by changes to local osmolarity as damaged cells release their cytosolic contents, leading to activation of a cytosolic phospholipase that metabolizes arachidonic acid precursors (Enyedi et al., 2013). It is not known whether muSCs would respond to changes of salts or metabolites in their local environment after an injury, but a number of cytosolic molecules found in myofibres have been shown to regulate muSC function including metabolic enzymes (Tsuchiya et al., 2020).

The injury method used will affect the muSC response. Several types of injury are commonly used to investigate muSC biology in mice. These cause different types of damage and therefore result in differing muSC responses (extent, duration, and resolution). A comparison of injury methods against the responses of muSCs revealed that freeze injury elicits a rapid and extensive activation and proliferation of muSCs that peaks at 1 month after injury (Hardy et al., 2016). In contrast, injection of myotoxic substances kills myofibres but leaves the extracellular matrix undamaged (Baghdadi and Tajbakhsh, 2018). Regeneration is a more prolonged affair in muscle damaged by myotoxins, with a peak number of proliferating muSCs occuring up to 3 months after injury (Hardy et al., 2016). One important difference between a freeze injury and myotoxin injury is that extracellular matrix and associated cells are unaffected in the case of the latter. Differences in muSC responses may reflect the importance of the extracellular matrix acting as a scaffold for regulating muSC biology. Mechanical cues are crucial for controlling muSC responses to injury with substrate stiffness dictating the rate of myoblast proliferation and differentiation (Engler et al., 2006). YAP/TAZ are activated in muSCs in response to reduced mechanical tension and play a crucial role in promoting muSC proliferation (Judson et al., 2012; Sun et al., 2017). Perturbation of mechanical tension due to disruption of the ECM by needle injury is therefore predicted to promote an upregulation of Yap activity. An intriguing link between YAP/TAZ activity and Notch has been identified whereby YAP activity in myofibres maintains Notch activity in adjacent muSCs (Esteves de Lima et al., 2016). Needle injury of muscle in the larval zebrafish myotome results in proliferation of muSCs in a Notch dependent manner. Although we did not measure tension experienced by muSCs after needle stick injury, the contraction of damaged myofibres suggests there is a reduced tension across the myotome. We note that over a period of approximately 48 h the myotome becomes smaller in response to larger injuries affecting 10 or more myofibres (Knappe et al., 2015). This could be because of a loss of tension required to maintain myotome size when damaged myofibres are removed by inflammatory cells. Therefore, if tension is reduced as a consequence of myofibre injury, elevated Yap/Taz activity might occur which could promote elevated Notch activity and hence muSC proliferation.

### Statistical Robustness for Exploring Muscle Satellite Cell Biology in Zebrafish

Zebrafish are increasingly used for investigating biological processes *in vivo* or for small molecule screening (North et al., 2007; Patton and Zon, 2008; Colanesi et al., 2012). Comparisons of cell biology between mammals and zebrafish are complicated by the rapid development of zebrafish larvae and that they have relatively few cells compared to a mouse. We observe an average of 18 cells responding to focal tissue injury in 8 dpf larvae. As there are relatively few units to compare between conditions (cells), it is important to ensure experiments are designed to have high power and a small effect size.

To increase statistical power and reduce the effect size we have several defined important variables that can affect the number of muSCs observed in response to tissue injury. These are: (1) injury size, (2) the interval at which muSCs are examined after injury, (3) the developmental stage of the animal.

In this study we show that we can induce a small injury leading to low variability in the number of muSCs responding. This is important as it reduces variability and so increases the power of the model (>80%), requiring fewer animals to detect a significance difference in response to a variable (*n* = 3, *n* = 5 animals required at 3 and 7 dpf, respectively). Our previous investigations of the injury response have revealed that muSCs migrate to injured myofibres from around 8 hpi and essentially have completed their migration by 24 hpi at both 3 and 7 dpf (Knappe et al., 2015). Proliferation of muSCs at the injury site continues after 24 hpi, therefore it is possible to measure this over a longer time. If injury size correlates with muSC proliferation, it is likely that differences in proliferation due to injury size will become more exaggerated over time. We used BrdU incorporation to measure proliferation from immediately after injury until 24 hpi. This measures all proliferation events and so represents a cumulative value, unlike other measures of proliferation, which count cells in a specific stage of the cell cycle. By increasing the number of cells that can be measured from a single animal there is more power of the statistical tests used. We have therefore benchmarked 24 hpi as the optimal stage for evaluating muSC responses to injury in zebrafish larvae by measuring BrdU incorporation. To determine the optimal stage to use for understanding muSC regulation during regeneration we compared the muSC response to injury of 3 and 7 dpf larvae relative to the contribution of these cells to myofibre growth. At both stages muSCs contribute to regeneration, but at 3 dpf there is also a contribution of muSCs to growth (Roy et al., 2017). We find that a larger sample size is needed for identifying statistical differences due to manipulation of Notch at both 3 and 7 dpf and that tests of significance are obtained at low power. There are several caveats for comparing muSC responses to injury in animals at different developmental stages. One, is that the relative injury is smaller at 7 dpf as the myotome is approximately 25% larger (Hinits et al., 2011). Secondly, there is heterogeneity between myoblast progenitors that may change as the muscle grows. Last, progenitor cells may have different cell cycle rates which will affect how quickly they respond to injury within a given time-frame. Nonetheless, we were able to show at high power with few samples, that Notch is required for muSC proliferation in response to injury. Although there was a lower power in our assays examining the importance of Notch in regulating muSC homeostasis we did see an intriguing elevated rate of proliferation of muSCs at the VM in uninjured animals. This suggests Notch also acts to prevent cycling of progenitor cells in an absence of injury, analogous to the mammalian muSC niche.

## Data Availability Statement

The original contributions presented in the study are included in the article/Supplementary Material, further inquiries can be directed to the corresponding author/s.

## Ethics Statement

The animal study was reviewed and approved by the Animal Welfare and Ethics Review Board of King’s College London. Experiments using animals were performed in accordance with the U.K. Animals (Scientific Procedure) Act 2012 and the European Union animal welfare Directive 2010/63/EU under project license PPL PBC5F9B13. Experimental design incorporated the principles of the 3Rs.

## Author Contributions

RK designed the project, planned the experiments, and wrote the manuscript. CD performed the experiments and analyzed the data. SS planned and performed the experiments, analyzed the data, and contributed to writing of the manuscript. All authors contributed to the article and approved the submitted version.

## Conflict of Interest

The authors declare that the research was conducted in the absence of any commercial or financial relationships that could be construed as a potential conflict of interest.

## Publisher’s Note

All claims expressed in this article are solely those of the authors and do not necessarily represent those of their affiliated organizations, or those of the publisher, the editors and the reviewers. Any product that may be evaluated in this article, or claim that may be made by its manufacturer, is not guaranteed or endorsed by the publisher.
