# Supplementary material for: Notch Signaling Regulates Muscle Stem Cell Homeostasis and Regeneration in a Teleost Fish
Source: Front Cell Dev Biol. 2021 Sep 28;9:726281. doi: 10.3389/fcell.2021.726281 (PMC8505724; doi:10.3389/fcell.2021.726281)
Supplement: Supplementary file 1 [file Data_Sheet_1.docx]

Notch signalling regulates muscle stem cell homeostasis and regeneration in a teleost fish

Sami H.A. Sultan^1^, Carlene Dyer^1,2^, Robert D. Knight^1*^

^1^ Centre for Craniofacial and Regenerative Biology, King’s College London, Guy’s Hospital, London SE1 9RT

^2^ William Harvey Research Institute, Barts and The London School of Medicine and Dentistry, Queen Mary University of London, Charterhouse Square, London EC1M 6BQ

Supplementary Material

**Supplementary figures**


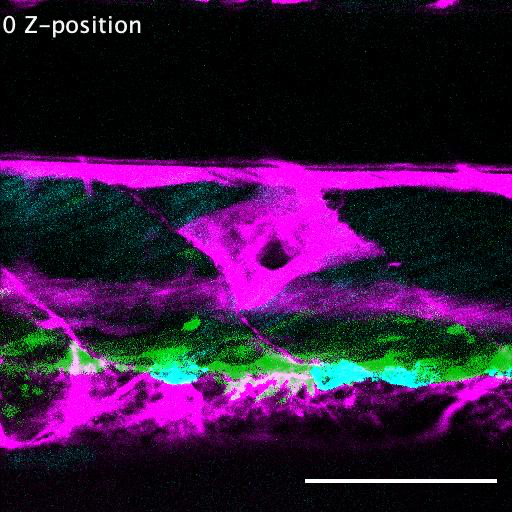


**Movie S1**

**
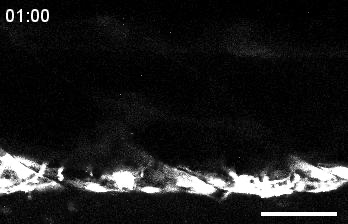
**

**Movie S2**

**
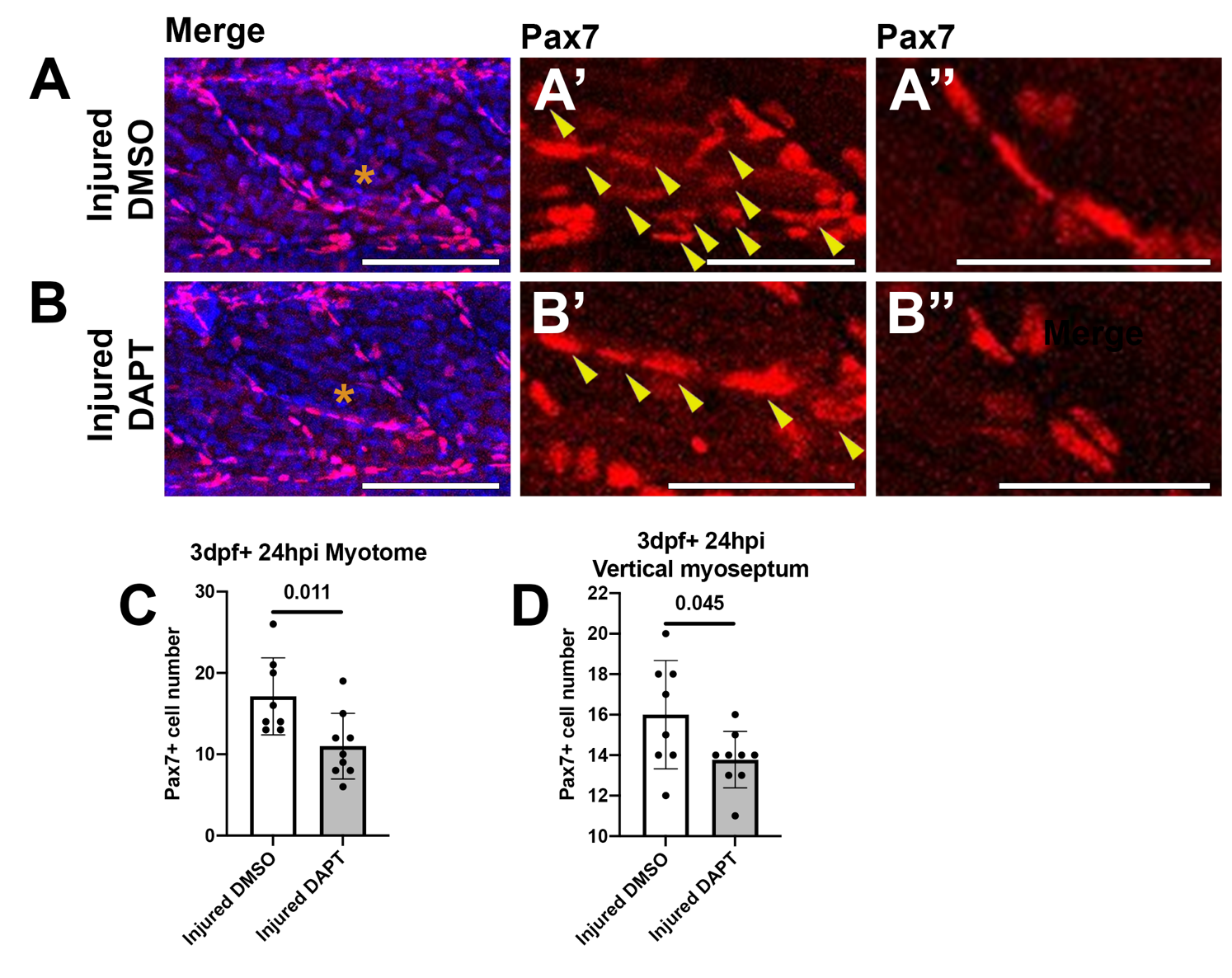
**

**Figure S1**

**
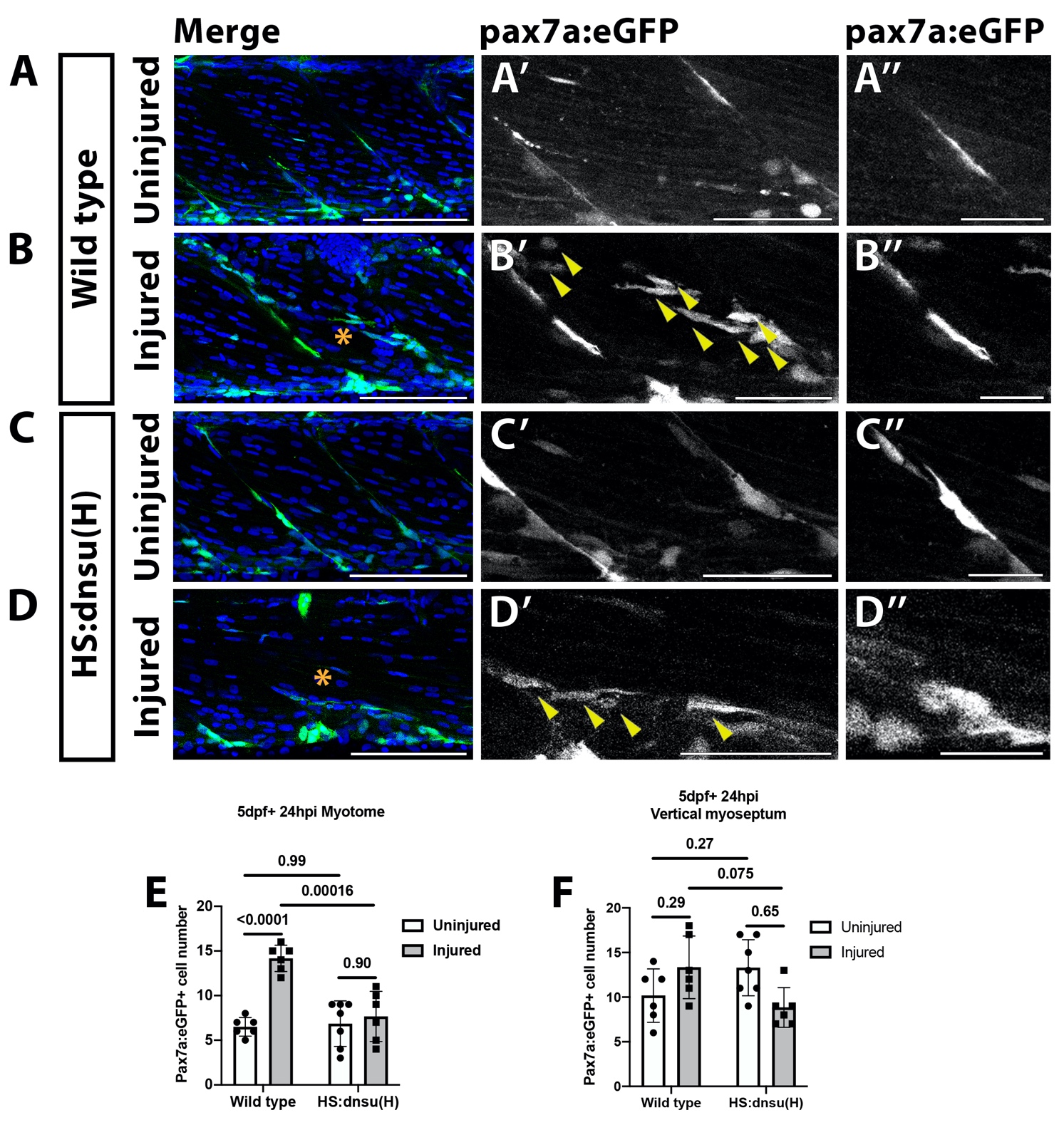
**

**Figure S2**

**
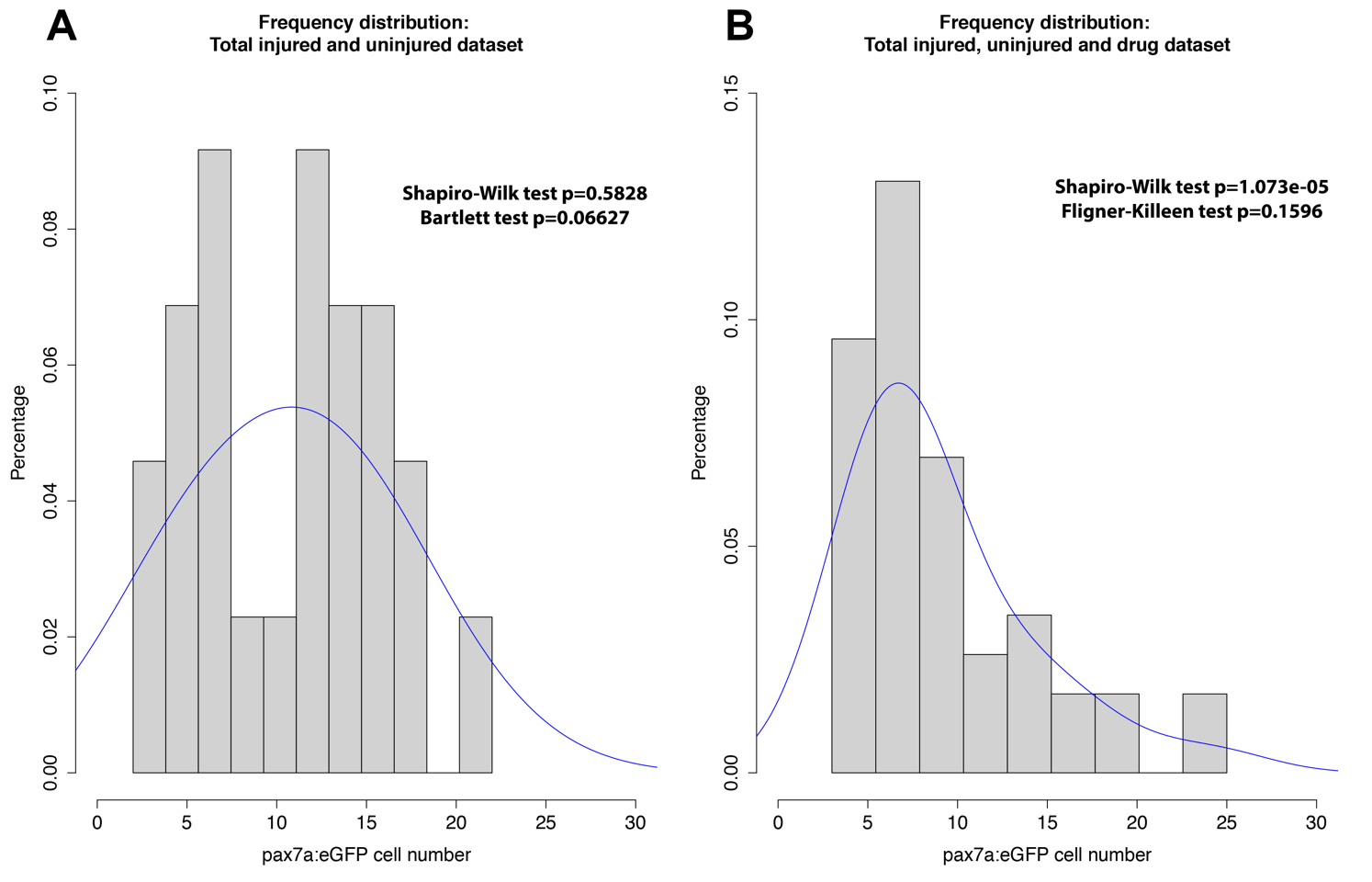
**

**Figure S3**

| Stage (dpf) | Injury (Y/N) | DAPT (µM) | DMSO (%) | Mean number of pax7a:eGFP+ cells | SD |
| --- | --- | --- | --- | --- | --- |
| 4 | N | 0 | 0 | 5.5 | 1.52 |
| 4 | Y | 0 | 0 | 11.67 | 1.86 |
| 8 | N | 0 | 0 | 8.33 | 4.68 |
| 8 | Y | 0 | 0 | 17.17 | 2.48 |
| 4 | N | 0 | 1 | 5.83 | 2.14 |
| 4 | Y | 0 | 1 | 14 | 1.61 |
| 4 | N | 100 | 0 | 7.5 | 1.87 |
| 4 | Y | 100 | 0 | 7.83 | 2.71 |
| 8 | N | 0 | 1 | 6.8 | 1.92 |
| 8 | Y | 0 | 1 | 17.33 | 6.19 |
| 8 | N | 100 | 0 | 5.5 | 1.52 |
| 8 | Y | 100 | 0 | 6.33 | 1.75 |

**Table S1**

| Factor/ interaction | Test | Cell type | Anatomical position | p-value |
| --- | --- | --- | --- | --- |
| Injury | ART ANOVA | Pax7a:eGFP | Myotome | 8.116E-07 |
| DAPT | ART ANOVA | Pax7a:eGFP | Myotome | 0.0010314 |
| Injury: DAPT | ART ANOVA | Pax7a:eGFP | Myotome | 0.0010134 |
| Injury | ANOVA | BrdU | Myotome | 0.00000416 |
| DAPT | ANOVA | BrdU | Myotome | 0.6495 |
| Injury: DAPT | ANOVA | BrdU | Myotome | 0.0387 |
| Injury | ART ANOVA | Pax7a:eGFP; BrdU | Myotome | 2.8875E-06 |
| DAPT | ART ANOVA | Pax7a:eGFP; BrdU | Myotome | 0.016057 |
| Injury: DAPT | ART ANOVA | Pax7a:eGFP; BrdU | Myotome | 0.046893 |
| Injury | ART ANOVA | % Pax7a:eGFP expressing BrdU | Myotome | 0.17714 |
| DAPT | ART ANOVA | % Pax7a:eGFP expressing BrdU | Myotome | 0.66174 |
| Injury: DAPT | ART ANOVA | % Pax7a:eGFP expressing BrdU | Myotome | 0.5496 |
| Injury | ANOVA | Pax7a:eGFP | Vertical myoseptum | 0.80971 |
| DAPT | ANOVA | Pax7a:eGFP | Vertical myoseptum | 0.13573 |
| Injury: DAPT | ANOVA | Pax7a:eGFP | Vertical myoseptum | 0.00908 |
| Injury | ANOVA | BrdU | Vertical myoseptum | 0.00457 |
| DAPT | ANOVA | BrdU | Vertical myoseptum | 0.43043 |
| Injury: DAPT | ANOVA | BrdU | Vertical myoseptum | 0.00327 |
| Injury | ANOVA | Pax7a:eGFP; BrdU | Vertical myoseptum | 0.3725 |
| DAPT | ANOVA | Pax7a:eGFP; BrdU | Vertical myoseptum | 0.1347 |
| Injury: DAPT | ANOVA | Pax7a:eGFP; BrdU | Vertical myoseptum | 0.0111 |
| Injury | ANOVA | % Pax7a:eGFP expressing BrdU | Vertical myoseptum | 0.698 |
| DAPT | ANOVA | % Pax7a:eGFP expressing BrdU | Vertical myoseptum | 0.198 |
| Injury: DAPT | ANOVA | % Pax7a:eGFP expressing BrdU | Vertical myoseptum | 0.531 |

**Table S2**

| Factor/ interaction | Test | Cell type | Anatomical position | p-value |
| --- | --- | --- | --- | --- |
| Injury | ART ANOVA | Pax7a:eGFP | Myotome | 3.80E-05 |
| DAPT | ART ANOVA | Pax7a:eGFP | Myotome | 6.46E-05 |
| Injury: DAPT | ART ANOVA | Pax7a:eGFP | Myotome | 1.33E-05 |
| Injury | ART ANOVA | Myog | Myotome | 0.00020317 |
| DAPT | ART ANOVA | Myog | Myotome | 0.13649489 |
| Injury: DAPT | ART ANOVA | Myog | Myotome | 0.74826122 |
| Injury | ANOVA | Pax7a:eGFP; Myog | Myotome | 0.753 |
| DAPT | ANOVA | Pax7a:eGFP; Myog | Myotome | 0.6 |
| Injury: DAPT | ANOVA | Pax7a:eGFP; Myog | Myotome | 0.255 |
| Injury | ANOVA | % Pax7a:eGFP expressing Myog | Myotome | 0.36808 |
| DAPT | ANOVA | % Pax7a:eGFP expressing Myog | Myotome | 0.06926 |
| Injury: DAPT | ANOVA | % Pax7a:eGFP expressing Myog | Myotome | 0.00134 |
| Injury | ANOVA | Pax7a:eGFP | Vertical myoseptum | 0.8564 |
| DAPT | ANOVA | Pax7a:eGFP | Vertical myoseptum | 0.4363 |
| Injury: DAPT | ANOVA | Pax7a:eGFP | Vertical myoseptum | 0.0351 |
| Injury | ART ANOVA | Myog | Vertical myoseptum | 0.54262 |
| DAPT | ART ANOVA | Myog | Vertical myoseptum | 9.82E-05 |
| Injury: DAPT | ART ANOVA | Myog | Vertical myoseptum | 0.54262 |
| Injury | ART ANOVA | Pax7a:eGFP; Myog | Vertical myoseptum | 0.68939 |
| DAPT | ART ANOVA | Pax7a:eGFP; Myog | Vertical myoseptum | 0.52393 |
| Injury: DAPT | ART ANOVA | Pax7a:eGFP; Myog | Vertical myoseptum | 0.61623 |
| Injury | ART ANOVA | % Pax7a:eGFP expressing Myog | Vertical myoseptum | 0.69046 |
| DAPT | ART ANOVA | % Pax7a:eGFP expressing Myog | Vertical myoseptum | 0.53869 |
| Injury: DAPT | ART ANOVA | % Pax7a:eGFP expressing Myog | Vertical myoseptum | 0.53626 |

**Table S3**

| Factor/ interaction | Test | Cell type | Anatomical position | p-value |
| --- | --- | --- | --- | --- |
| Injury | ANOVA | Pax7a:eGFP | Myotome | 6.38E-05 |
| dnsu(h) | ANOVA | Pax7a:eGFP | Myotome | 0.00232 |
| Injury: dnsu(h) | ANOVA | Pax7a:eGFP | Myotome | 0.000603 |
| Injury | ANOVA | Pax7a:eGFP | Vertical myoseptum | 0.5337 |
| dnsu(h) | ANOVA | Pax7a:eGFP | Vertical myoseptum | 0.6535 |
| Injury: dnsu(h) | ANOVA | Pax7a:eGFP | Vertical myoseptum | 0.00477 |

**Table S4**

**Supplementary figure legends**

**Movie S1. Evans blue labelling co-localised with a second harmonic signal from myofibres throughout the extent of the injury field.**

7dpf pax7a pax7a:eGFP larvae were submerged in 0.1% Evans blue for 1h, injured and then imaged on a multiphoton microscope. Visualisation of Evans blue in damaged myofibres (magenta), second harmonic generation (SHG) in myofibres (cyan) and pax7a:eGFP (muSC; green) reveals extensive co-localisation of the Evans Blue and SHG signal.

Scale bar 100µm.

**Movie S2. muSC response to single myotome injury.**

Injured 3 dpf pax7a:eGFP^+^ larvae were imaged by multiphoton microscopy from 1-16.83 hpi with 10 minute intervals between frames. Acquisition encompassed the entire injured myotome and z-stack projections reveal the migration of muSCs in response to injury.

Scale bar 50µm. Time stamp in minutes post injury.

**Figure S1. Notch inhibition by DAPT treatment results in fewer Pax7+ muSCs responding to muscle injury.**

Projections of confocal stacks (**A-B**) of the myotome (**A’-B’**) and vertical myoseptum (**A’’-B’’**) of injured 3 dpf larvae. Larvae were treated with 1% DMSO (**A**) or 100 µM DAPT (**B**) after injury, fixed at 24 hpi and labelled with anti-Pax7. MuSCs (yellow arrowheads) are recruited to the injury (asterisk) site (**A’-B’**). The number of cells expressing Pax7 were counted in the myotome (**C**) and vertical myoseptum (**D**) of animals treated with DMSO or DAPT. There were significantly fewer Pax7+ muSCs in the presence of DAPT compared to DMSO treated control animals in the myotome (p<0.05; **C**) and vertical myoseptum (p<0.05; **D**). Tests for significant differences were performed by Student’s t-test (n=8-9 animals per condition). Error bars display standard deviation, and values above comparison bars indicate significance (p-values).

Scale bars: 100 µm (**A-B**), ﻿50 µm (**A’-B’, A’’-B’’**).

**Figure S2. Expression of a dominant negative version of the Suppressor of hairless results in fewer muSCs responding towards muscle injury.**

Projections of confocal stacks (**A-D**) of the myotome (**A’-D’**) and vertical myoseptum (**A’’-D’’**) in injured 5 dpf larvae. Larvae were heat shocked for 1h, injured and fixed 24 hpi. Pax7a:eGFP expressing cells were detected in larvae which were wild type (**A-B**) or expressing heat shock dominant negative suppressor of hairless (HS:dnsu(h)) (**C-D**). MuSCs (yellow arrowheads) are recruited to the injury (asterisk) site (**B’-D’**). Scale bars: 100 µm (**A-B**), ﻿50 µm (**A’-B’, A’’-B’’**). The number of cells expressing pax7a:eGFP were counted in the myotome (**E**) and vertical myoseptum (**F**) of wild type and HS:dnSu(h) expressing larvae. Following injury, there were significantly fewer pax7a:eGFP+ muSCs in the presence of dnSu(h) compared to wild type control animals in the myotome (p<0.05; **E**). There were no significant changes to the number of pax7a:eGFP expressing cells in the vertical myoseptum (p<0.05; **F**). Significant differences were tested by 2-way ANOVA (n=25) with Tukey’s HSD post-hoc test. Error bars display standard deviation, and values above comparison bars indicate significance (p-values).

Scale bars: 100 µm (**A-C**), ﻿50 µm (**A’-C’, A’’-C’’**).

**Figure S3. Normality and scedasticity.**

Histograms with density curves illustrating the frequency distribution of pax7a:eGFP^+^ cell number across the datasets analysed. Datasets were grouped as follows: animals at 4 and 8 dpf with or without injury (**A**, n=24); animals at 4 and 8 dpf, with or without injury, treated with DAPT or DMSO (**B**, n=47). The Shapiro-Wilk test was used to assess for normality. To assess scedasticity a Bartlett test (**A**) or Fligner-Killeen test (**B**) was used.

**Table S1. Summary of experimental datasets.**

Experimental parameters for each data set have been listed showing the mean number of pax7a:eGFP+ muSCs and standard deviation (SD). Parameters include the age of the larvae (4 or 8 dpf), if the larvae were injured (yes or no) and treated with DAPT (0 µM or 100 µM) or DMSO (0% or 1% v/v).

**Table S2. Results from 2-way ANOVA testing the importance of injury, DAPT and interaction effects on the number of proliferating muSCs.**

A 2-way ANOVA was used to test for differences in the number of GFP, BrdU+ and GFP+/ BrdU+ cells in the myotome and vertical myoseptum of uninjured and injured pax7a:egfp larvae treated with DMSO or DAPT. Non-parametric data was transformed by ART. P-values indicate significance.

**Table S3 Results from 2-way ANOVA testing the importance of injury, DAPT interaction effects on the number of muSCs expressing myogenin.**

A 2-way ANOVA was used to test for differences in the number of GFP+, Myog+ and GFP+/ Myog+ cells in the myotome and vertical myoseptum of uninjured and injured pax7a:egfp larvae treated with DMSO or DAPT. Non-parametric data was transformed by ART. P-values indicate significance.

**Table S4. Results from 2-way ANOVA results testing the importance of injury, HS:dnsu(h) expression and interaction effects on the number of muSCs.**

A 2-way ANOVA was used to test for differences in the number of GFP+ muSCs in the myotome and vertical myoseptum of uninjured and injured pax7a:egfp with and without HS:dnSu(h) expression. P-values indicate significance.

**Supplementary methods**

**The experimental design**

To detect statistically significant differences between samples, pairwise two-tailed tests were performed with an assumption of two independent samples in which the null hypothesis ($H_{0}$) states there is no difference between the means ($\mu0,\mu1$) and the alternative hypothesis ($H_{1}$) assumes a difference between the means:

$$H_{0}:\mu_{0}=\mu_{1}$$

$$H_{1}:\mu_{0}{\neq\mu}_{1}$$

The population has a unique distribution defined by mean and the variance ($\Sigma^{2})$:

$$(\mu_{0}, \Sigma_{0}^{2})$$

$$(\mu_{1}, \Sigma_{1}^{2})$$

**Assessing normality and scedasticity**

Before conducting pairwise and power analysis, normality and homoscedasticity (equal variance) was assessed. Normality was assessed using the Shapiro–Wilk test function. If the results of the Shapiro–Wilk test was greater than 0.05 (p>0.05) then normality was assumed. A density histogram was produced to visualise and assess the data for normality and variance (Fig S1). If the dataset was parametric, the Bartlett test was used to determine scedasticity, whereas if the dataset was non-parametric the Fligner-Killeen test was used. If the results for either Bartlett or Fligner-Killeen test was greater than 0.05 (p>0.05) then the assumption was that data has equal variance across the different conditions.

**Error**

Power and ideal sample size of an experiment are dictated by the probability of a type I or type II error occurring. A type I error, also referred to as α, is the probability of rejecting the null hypothesis when the null hypothesis is true (false positive) (Banerjee et al. 2009). The α-level was set to 0.05, which defines the point of significance. A type II error, also referred to as beta (β), is the probability of accepting the null hypothesis when the null hypothesis is false (false negative) (Banerjee et al. 2009). Therefore, as power is the probability of rejecting the null hypothesis when the null hypothesis is false, power can be defined as the probability of not making a type II error (1-β; Cohen, 1992). When calculating the optimal sample size for future experiments, power was set to 0.8 (80%) which confers a probability of making a type II error as 0.2 (20%).

**i) Calculating the probability of a type I error**

**Student’s t-test**

A Student’s t-test calculates a t- or z- statistic to assess differences in means from two samples. The assumption for this test is that the data is normally distributed with equal variance. The t-statistic can be defined as:

$$t= \frac{(\bar{x}-\mu)}{\frac{S_{\sigma}}{\sqrt{n}}}$$

(Lachin 1981; Ogston et al. 1991)

The sample mean ($\mu$), sample standard deviation ($S_{\sigma}$) and sample size ($n$) of the test are used to calculate the t-value ($t$). Using a t-table, the calculated t-values are used to identify the associated p-value to the correct degrees of freedom ($n-1$). Due to the low sample number in the datasets used for this study, a t-statistic was used instead of a z-statistic (t-approximation). Student’s t-test were performed using a t.test (R: t.test(x, y, alternative = two.sided, var.equal= TRUE)) in which x and y define the two means. The same function was used to calculate the 95% confidence intervals for each mean (R: t.test(x/y, conf.level = 0.95)). The null hypothesis was rejected and the alternative hypothesis is accepted if the p-value is below the alpha critical level (p<0.05).

**Wilcoxon Mann Whitney rank sum test**

Data which was not normally distributed (non-parametric) was analysed using a ﻿Wilcoxon Mann-Whitney rank- sum (WMW) test. The WMW test ranks the changes in sample distributions in which m and n are the sample sizes for samples i and j respectively:

﻿W = $\Sigma_{i-1}^{m}\Sigma_{j-1}^{n}$p(Y_j_ −X_i_)

(Mollan et al. 2019)

In order to detect any difference in the two distributions (i and j), the WMW test sequentially examines each data point from one distribution (Y_j_) to the data points in the other distribution (X_i_). If Y_j_ is greater than X_i_, the W value is defined as 1 (p(Y_j_ −X_i_) = 1). If this criteria is not met (Y_j_ is not greater than X_i_), the W value is set to 0 (Mollan et al. 2019). Therefore, the WMW test quantifies every instance Y_j_ is greater than X_i_ generating an overall W value (W-statistic). Using a W-table (also referred to as u-table), the W critical value and p-value at a specified alpha level and sample size are identified. If W< W critical value at an alpha level of 0.05 we reject the null hypothesis concluding there is a statistical difference in the distributions. WMW tests were performed with an assumption of a 2-tailed distribution in which x and y define the means for the two populations (R: wilcox.test(x, y, alternative = two.sided)). The same function was used to calculate the 90% confidence intervals for each mean (R: wilcox.test(x/y, conf.level = 0.90)). Due to the low sample size and ties within the data, a normal approximation was used on the ranked data with a maximum confidence interval of 90% (Hollander, A. Wolfe, and Chicken 2013).

**Multi-parametric analysis**

In order to assess the difference in means between two or more factors, an Analysis of variance (ANOVA) was conducted. An ANOVA measures the effects of multiple factors in addition to determining any interactions between factors. Data which is parametric was analysed using ANOVA (R: aov). Data which was non-parametric was analysed using a rank test. Aligned Rank Transformation (ART) aligns the data before determining the rank (Wobbrock et al. 2011). Standard ranking techniques used to analyse non-parametric data lead to inaccuracies when determining interactions effects. ART eliminates this problem by first estimating the effect of factors (both main and interaction effects) as marginal means with all other effects removed (aligning) before ranking the data, accurately determining the effect of each factor (Wobbrock et al. 2011). Data showing a non-parametric distribution with equal variance was transformed and ranked using the ARTool package (R: ART) then ranked data analysed by ANOVA.

When conducting sequential pairwise analysis between conditions on the same dataset, a correction for multiple comparisons were applied to minimise the occurrence of type I and II error. For parametric data, a one-way ANOVA with Tukey’s honest significant difference (HSD) post-hoc test was applied, which is appropriate for test with small sample sizes (Barnette and McLean 1999). Using the base R: aov to run an ANOVA, a Tukey’s HSD multiple comparisons was conducted:

R: TukeyHSD(model, conf.level=0.95, alpha= 0.05)

The model defines the results from an ANOVA, and the confidence interval and alpha level are set to 0.95 and 0.05, respectively.

For non-parametric data, a Kruskal-Wallis one way AOVA with Dunn’s post-hoc test was applied with the Benjamini-Hochberg correction. This test is suitable for non-parametric data (Dinno 2015), accounting for multiple comparisons by assessing the false discovery rate (Benjamini and Hochberg 1995). Using the dunn.test package (Dinno 2017), the R: dunn.test function was used:

R: dunn.test(x , g, method = "bh", alpha = 0.05)

The cell population (x) and conditions (g) were defined and a Benjamini-Hochberg (bh) correction was applied. The alpha level was set to 0.05.

**ii) Calculating the probability of a type II error**

**Student’s t-test**

A Student’s t-test was used to calculate power and ideal sample size for data showing a normal distribution and equal variance. Sample size (N) affects the power of the analysis as it dictates total variance ($\Sigma^{2}$) of the data in combination with the standard deviation ($\sigma$).

$N(\Sigma^{2}= \frac{\sigma^{2}}{N})$.

When using a t or z-statistic to calculate significance, the ideal sample size avoids both type I and II errors and therefore simultaneously satisfies the following expressions:

$$P\left( Z>Z_{\alpha/2} \right)= \alpha| H_{0} is true$$

$$P\left( Z>Z_{\alpha/2} \right)= 1-\beta| H_{1} is true$$

These expressions require that the probability (P) of a given value of Z (the t or z statistic) being greater than Z at a given alpha-level ${(Z}_{\alpha/2}),$ is equivalent to alpha ($\alpha$) when $\alpha$=0.05 if the null hypothesis is true and the means are not significantly different. In addition, the probability of a given value of Z being greater than Z at a given alpha-level ${(Z}_{\alpha/2})$ is equivalent to 1-$\beta$ where $\beta$ is the probability of a type II error when the alternative hypothesis is true. This means that the ideal sample size is one which accepts the null hypothesis when it is true reducing the probability of a type I error, or rejects the null hypothesis and accepts the alternative hypothesis when the null hypothesis is false, reducing the probability of a type II error. The Student’s t-test can be used to calculate both sample size and power when the following conditions are met:

1. The proportion $(Q)$ of samples in each group is defined as follows:

$$Q_{e(experimental)}+Q_{c(control)}=1$$

2. Variance of individual samples $(\sigma^{2})$ is equal between groups:

$$\Sigma_{0}^{2}= \Sigma_{1}^{2}= \frac{\sigma^{2}(Q_{e}^{-1}+Q_{c}^{-1})}{N}$$

3. The experimental ($v_{e}$) and control ($v_{c}$) means (${v_{e}-v_{c}=\mu}_{1}$) are normally distributed.

Ideal sample size ($N$) and power ($Z_{\beta}$) can be calculated by the following expressions:

$$N= \frac{\sigma^{2}(Q_{e}^{-1}+Q_{c}^{-1}){(Z_{\alpha}+Z_{\beta})}^{2}}{\mu_{1}^{2}}$$

$$Z_{\beta}=\frac{\left| \mu_{1} \right|\sqrt{N}-Z_{\alpha}\sigma\sqrt{(Q_{e}^{-1}+Q_{c}^{-1})}}{\sigma\sqrt{(Q_{e}^{-1}+Q_{c}^{-1})}}$$

(Lachin 1981)

In the present study we first aimed to calculate the retrospective power of our analysis at an alpha level of 0.05. Subsequently, we used the datasets produced to identify the ideal sample size needed to achieve a power of 80% for future experiments. Using a t-approximation (t-table and t-value) for the Z-value, the $Z_{\alpha}$ and $Z_{\beta}$ value can be obtained for a two-tailed analysis at an $\alpha$-level of 0.05 and power of 80% (probability of a type I error (p)=0.02) to the appropriate degrees of freedom ($n-1$). In order to calculate both power and ideal sample size, the R: power_t_test function was used (MESS package). When calculating power, the known experimental sample size is included and the power is set to NULL. However, when calculating the ideal sample size at a power of 80%, power is set to 0.8 (80%) and samples size is set to NULL.

(R: power_t_test(n = (NULL or sample size), delta = , sd = , sig.level = 0.05, power = (NULL or 0.8), ratio = , sd.ratio = , type= two.sample, alternative = two.sided, df.method = classical)

By setting one of these two parameters (power or sample size) to NULL, the R: power_t_test function will calculate the missing parameter.

**Wilcoxon Mann Whitney (WMW) rank sum test**

The WMW test was used when data was non-parametric to calculate the power and ideal sample size. The wmwpow package (Shieh et al, 2006) was used to calculate power using the Wilcoxon-Mann-Whitney rank-sum test assuming a two-tailed (sides = "two.sided") normal distribution (dist = "norm") with an $\alpha$-level of 0.05 (alpha = 0.05). This assumes the sample size for both groups (n and m) and the probability of the two distributions are different (p; 1- the probability of a type I error).

(R: shiehpow(n = , m = , p = , alpha = 0.05, dist = "norm", sides = "two.sided")

The WMWssp package (Happ, Bathke, and Brunner 2019) was used to calculate the required sample size to achieve 80% power by running a simulation on the data provided. Sample size was calculated to achieve 80% power (power = 0.8) at an $\alpha$-level of 0.05 (alpha = 0.05) in which x and y define the means of the two samples and t defines how the calculated ideal sample size (n) is allocated between conditions (set to 0.5 so evenly distribute samples between conditions)

(R: WMWssp(x, y, alpha = 0.05, power = 0.8, t = 0.5).

**Calculating the effect size**

The results of the test statistic are dependent on various conditions, one of which is the effect size. The effect size is the measure of the difference of sample means, thus the larger the effect size, the higher the power and significance of the statistical test (Cohen 1988). To determine the relationship between effect size and power the Cohen’s d coefficient was calculated. This coefficient was calculated by dividing the difference in sample means ($\Delta\bar{\mu}$) by the standard deviation as follows:

$$Cohen^{'}s d= \frac{\left| \Delta\bar{\mu} \right|}{\bar{\sigma}}$$

In order to account for a difference in scedasticity of the two means $\left( \sigma\right)$ a pooled standard deviation (SD) was used which is calculated from the standard deviation of both samples.

$$Cohen^{'}s d= \frac{\left| \Delta\bar{\mu} \right|}{SD\mathrm{pooled}}$$

Where ${SD}_{pooled}={\surd((\sigma}_{1}^{2}+\sigma_{2}^{2})/2)$

**Supplementary references**

Banerjee, Amitav, UB Chitnis, SL Jadhav, JS Bhawalkar, and S Chaudhury. 2009. “Hypothesis Testing, Type I and Type II Errors.” *Industrial Psychiatry Journal* 18 (2): 127. https://doi.org/10.4103/0972-6748.62274.

Barnette, J Jackson, and James E McLean. 1999. “The Tukey Honestly Significant Difference Procedure and Its Control of Type I Error Rate.” *Report*.

Benjamini, Yoav, and Yosef Hochberg. 1995. “Controlling the False Discovery Rate: A Practical and Powerful Approach to Multiple Testing.” *Journal of the Royal Statistical Society: Series B (Methodological)*. https://doi.org/10.1111/j.2517-6161.1995.tb02031.x.

Cohen, Jacob. 1988. “Statistical Power Analysis for the Social Sciences (2nd Ed.).” *Hillsdale NJ: Erlbaum.*

Cohen, Jacob. 1992. “Statistical Power Analysis.” *Current Directions in Psychological Science* 1 (3): 98–101.

Dinno, Alexis. 2015. “Nonparametric Pairwise Multiple Comparisons in Independent Groups Using Dunn’s Test.” *Stata Journal*. https://doi.org/10.1177/1536867x1501500117.

Dinno, Alexis. 2017. “Dunn.Test: Dunn’s Test of Multiple Comparisons Using Rank Sums.” *R Package Version 1.3.5*.

Happ, Martin, Arne C. Bathke, and Edgar Brunner. 2019. “Optimal Sample Size Planning for the Wilcoxon-Mann-Whitney Test.” *Statistics in Medicine* 38 (3): 363–75. https://doi.org/10.1002/sim.7983.

Hollander, Milles, Douglas A. Wolfe, and Eric Chicken. 2013. “Wilcoxon.” In *Nonparametric Statistical Methods*.

Lachin, John M. 1981. “Introduction to Sample Size Determination and Power Analysis for Clinical Trials.” *Controlled Clinical Trials* 2 (2): 93–113. https://doi.org/10.1016/0197-2456(81)90001-5.

Mollan, Katie R., Ilana M. Trumble, Sarah A. Reifeis, Orlando Ferrer, Camden P. Bay, Pedro L. Baldoni, and Michael G. Hudgens. 2019. “Exact Power of the Rank-Sum Test for a Continuous Variable.” *ArXiv*, 1–15. http://arxiv.org/abs/1901.04597.

Ogston, S. A., S. Lemeshow, D. W. Hosmer, J. Klar, and S. K. Lwanga. 1991. “Adequacy of Sample Size in Health Studies.” *Biometrics* 47 (1): 347. https://doi.org/10.2307/2532527.

Wobbrock, Jacob O., Leah Findlater, Darren Gergle, and James J. Higgins. 2011. “The Aligned Rank Transform for Nonparametric Factorial Analyses Using Only ANOVA Procedures.” *Conference on Human Factors in Computing Systems - Proceedings*, 143–46. https://doi.org/10.1145/1978942.1978963.
